# Odour preferences of children and adults across four diverse cultures in Africa, Asia, Europe, and Oceania

**DOI:** 10.3758/s13423-026-02984-7

**Published:** 2026-10-26

**Authors:** Marta Kowal, Piotr Sorokowski, S. Craig Roberts, Marina Butovskaya, Syed Imran Ali Shah, Wiktoria Jędryczka, Kateřina Roberts, Oana David, Michal Pieniak, Thomas Hummel, Agnieszka Sorokowska

**Affiliations:** 1 The International Institute for Mental Health, Babes-Bolyai University, Cluj-Napoca, Romania; 2 IDN Being Human Lab - Institute of Psychology, University of Wrocław, Wrocław, Poland; 3 School of Psychology, University of Stirling, Stirling, UK; 4 Institute of Ethnology and Anthropology, Russian Academy of Sciences, Moscow, Russia; 5 Department of Biochemistry, Azra Naheed Medical College, Faculty of Medical Sciences, Superior University, Lahore, Pakistan; 6 Institute of Psychology, University of Wrocław, Wrocław, Poland; 7 Department of Zoology, Faculty of Science, Charles University, Prague, Czech Republic; 8 Smell and Taste Clinic, Department of Otorhinolaryngology, Technical University of Dresden, Dresden, Germany

**Keywords:** Odour pleasantness, Smell, Odour universality, Sniffin’ Sticks, Cross-cultural

## Abstract

Odour perception plays a crucial role in various processes, yet the extent to which odour preferences are universal or shaped by cultural and ecological contexts remains debated. While some studies report substantial cross-cultural agreement in odour pleasantness, others highlight considerable variability. To address this question from a novel perspective, we collected data from 394 children and adults (aged 3–81) across four distinct cultures (Hadza, Pakistani, Dani, and Scottish). Participants rated the pleasantness and familiarity of 11 odours (Fish, Garlic, Butter, Ginger, Thyme, Cinnamon, Banana, Peach, Strawberry, Jasmine, Butanol). We observed both variability and agreement in odour pleasantness ratings across cultures, but overall, there was a similar level of agreement across age groups within each culture. Furthermore, the most pleasant odours (Jasmine, Strawberry, Peach) were rated more consistently across cultures than the most unpleasant ones (Fish, Garlic, Butter). Finally, familiarity was strongly and positively associated with pleasantness. These findings suggest that while some universal consensus in odour evaluation exists, ecological and cultural influences, as well as individual differences, may all play a substantial role in shaping olfactory perception.

**Supplementary Information:**

The online version contains supplementary material available at https://doi.org/10.3758/s13423-026-02984-7.

## Introduction

Given the crucial role of odours in food evaluation, hazard avoidance, and social communication (Stevenson, 2010), understanding the factors that shape odour perception is a key topic in olfactory research. Is human odour perception universal, determined by the chemical properties of odorants, or largely variable, learned through experience and context? Theoretical models generally distinguish between two primary frameworks. The universalist position suggests that hedonic responses are partially innate, driven by the physicochemical properties of odorants and evolutionarily conserved olfactory receptors (Khan et al., 2007). In contrast, the relativistic position proposes that preferences are largely acquired through associative learning. This learning can occur at the cultural level, where shared dietary and environmental exposures shape group preferences, or at the idiosyncratic level, driven by an individual’s unique personal history and specific emotional memories (Herz, 2016). Importantly, these two positions are likely not mutually exclusive, but jointly shape olfactory perception: innate biological predispositions may establish a baseline of hedonic valence, which is subsequently fine-tuned or even overridden by the accumulation of cultural and personal experiences over the lifespan (Poncelet et al., 2010).

Disentangling the relative weight of these influences is complex, but several cross-cultural studies have attempted to address this issue. Arshamian et al. (2022) tested 225 individuals from nine diverse cultures, ranging from the hunter-gatherer Maniq people of Asia to urban-dwelling Americans, asking them to rank monomolecular odorants by pleasantness; they found substantial cross-cultural consistency in odour rankings. A study by Drnovsek et al. (2025) supports this view: adults from 16 different urbanised regions showed significant agreement in pleasantness ratings of different monomolecular odorants. In line with this, results from probably the largest citizen-science project, the “Smell Survey”, with over 1.42 million respondents, found that individual differences in odour pleasantness ratings were much larger than differences between countries (Wysocki et al., 1991); this pattern would suggest that odour perception is primarily driven by individual-level factors, which might include biological factors (e.g. genetic variation in olfactory receptors), idiosyncratic personal histories, and unique emotional associations. Consequently, the influence of cultural variation in odour perception may be comparatively small.

However, other work has emphasised the role of cultural influences in odour perception. For instance, Sorokowski et al. (2024) compared pleasantness ratings for 15 odour samples in 582 people across five ecologically diverse cultures, including Hadza hunter-gatherers from Tanzania, Tsimané forager-horticulturalists from Amazonia, Yali horticulturalists from the Papuan highlands, and industrialised Poles and Malaysians. Although they found some cross-cultural similarities in odour assessments, they also observed cross-cultural variability in odour pleasantness ratings and concluded that cultural influences on odour perception are undeniable.

Other studies have focused on the issue of universality by comparing odour preferences among children, whose cultural exposure to smells is more limited (e.g. Oleszkiewicz et al., 2022; Schaal et al., 1996). For instance, Schaal et al. (1996) collected data from children aged 6–12 years from four countries (France, Canada, Indonesia, and Syria); their results revealed a distinct asymmetry: while participants across cultures largely agreed on the evaluation of unpleasant odours (similarity), ratings for pleasant odours showed significantly higher divergence between groups (variability). Conversely, Oleszkiewicz et al. (2022) studied 510 children (aged 5–8) in 18 countries, and concluded that pleasantness ratings were relatively consistent and primarily driven by odour qualities.

To shed more light on the issue of cultural variability in odour perception, we approached it from another perspective, hypothesising that a cross-cultural comparison of odour preferences among both children and adults may provide valuable insight. Although chemosensory learning begins early, potentially even prenatally (Forestell, 2024; Mennella et al., 2001), the cumulative effect of cultural exposure (e.g. decades of dietary habits and social reinforcement) is substantially greater in adults than in children (Grusec & Hastings, 2015). Therefore, if odour preferences are significantly influenced by culture, variability in odour ratings should be smaller in younger age groups and progressively greater in older ones. Analysing data across different age groups using the same odour samples and procedures enables us to disentangle the influence of culture from innate predispositions, while also holding within-culture environmental conditions constant. Strong agreement in odour preferences across age groups and cultures would suggest that culture may have a modest impact on odour liking. If, however, odour pleasantness ratings differ across age groups or vary across cultures, it would signal a stronger impact of cultural experiences on olfactory preferences.

To our knowledge, however, except for research on the importance of olfaction in daily life (Sorokowski et al., 2023), there have been no cross-cultural studies involving both children and adults from diverse cultural backgrounds. The few studies that have compared odour perception among both children and adults did so within single cultures and yielded inconclusive findings (e.g. Cameron et al., 2024; Schmidt & Beauchamp, 1988; Stevenson et al., 2007).

Another interesting question concerns the universality of perception of unpleasant versus pleasant odours. From an evolutionary perspective, one would expect that hedonic ratings of odours may be more similar when smelling more unpleasant than pleasant odours. Unpleasant odours can signal a threat of potential transmission of pathogens (e.g. from rotten food or animal waste) and, consistent with Behavioral Immune System theory (Schaller & Park, 2011), should thus trigger innate aversion and disgust. Disgust reactions to unpleasant odours can act as an “early warning”, reducing the likelihood of consuming spoiled food or approaching a source of potential contamination (Stafford et al., 2018; Stevenson, 2010). Indeed, some studies have found empirical evidence for higher universality of assessments of unpleasant odours than pleasant ones (e.g. Schaal et al., 1996; Schmidt & Beauchamp, 1988). We expected to replicate these findings and find higher agreement in ratings of unpleasant than pleasant odours.

However, familiarity may also play a key role in pleasantness assessments. In general, familiarity tends to increase liking (Ojeda et al., 2021; Torrico et al., 2019). In the domain of diet, repeated exposure and dietary prevalence often predict taste preferences (Aldridge et al., 2009). For instance, Sorokowska et al. (2017) found that the Hadza preferred tastes that are common in their diet, and Pieniak et al. (2022) showed that Cook Islanders’ liking for sour foods was related to their familiarity with this taste. Similar familiarity–liking links have been reported for odours (Delplanque et al., 2015; Ferdenzi et al., 2013; Seo et al., 2008). Consistent with this, Sorokowski et al. (2024) found familiarity to be the strongest predictor of odour pleasantness across the five cultures in their study. We therefore predicted a positive association between familiarity and odour pleasantness ratings.

Furthermore, context effects represent an additional factor that may shape hedonic responses to odours. Research has demonstrated that the verbal label provided alongside an odorant can substantially alter its perceived pleasantness, even when the chemical stimulus remains the same (Herz & von Clef, 2001). Neuroimaging evidence has similarly shown that odour-congruent contextual cues modulate hedonic responses at the neural level (Araujo et al., 2005). Such effects may be especially pronounced in cross-cultural and food-related contexts, where the same odorant can carry markedly different associations depending on cultural background and prior experience. In the present study, most of the odours were food-related, and it is therefore plausible that differences in contextual framing across cultures may contribute to variability in pleasantness ratings.

To test the aforementioned predictions, we conducted a cross-cultural study on children’s and adults’ odour preferences. The study was conducted among culturally, geographically, and environmentally diverse populations, including representatives of the Western world inhabiting an urban area (Scots from the city of Stirling), members of a partly hunter-gatherer community from the Tanzanian savannah (Hadza), Pakistanis from the peri-urban areas of Lahore, and an indigenous Papuan community (Dani) living in mountainous forests. We selected 11 odorants (Fish, Garlic, Butter, Ginger, Thyme, Cinnamon, Banana, Peach, Strawberry, Jasmine, and Butanol) varying in potential pleasantness. Each participant rated each odorant’s pleasantness and familiarity. A summary of our hypotheses is presented in Table 1.

**Table 1:** A summary of the hypotheses and results

Hypotheses:		Evidence to support?	Conclusion
H1:	Members of four distinct cultures rate odour pleasantness similarly	**x/✔**	High cross-cultural agreement was observed in the rank order of odours, but significant cross-cultural differences in pleasantness absolute ratings and consensus suggest that cultural factors play a substantial role in shaping odour preferences
H2:	Odour pleasantness ratings are more consistent (less variable) in younger than in older age groups	**x**	No overall age effect on pleasantness ratings
H3:	The most unpleasant odours are rated more similarly across cultures than the most pleasant odours	**x**	The most pleasant odours were rated more consistently than the most unpleasant ones
H4:	Familiarity is positively related to odour pleasantness ratings	**✔**	Higher odour familiarity was associated with higher pleasantness ratings

## Method

The study procedure complied with the Declaration of Helsinki and was approved by the authors’ institutional ethics committees, the local Dani community in West Papua, and the Tanzanian Commission for Science and Technology (COSTECH). All data, R scripts, and Supplementary Materials (SM) are publicly available on the OSF (https://osf.io/54dyr).

### Power analysis

Since some data were collected among hard-to-reach populations (i.e. Hadza and Dani), we aimed to obtain the largest possible sample rather than specifying a predetermined sample size. Therefore, instead of conducting an a priori power analysis, we performed a sensitivity analysis using the collected data. We ran 1,000 Monte Carlo simulations, assuming 394 participants, 4,334 odour ratings, and four predictors with the following average standardised coefficients: β₁ = 0.019, β₂ = 0.206, β₃ = 0.457, and β₄ = 0.246. The results indicated that we had nearly 100% power to detect the observed average effect sizes for culture, type of odour, and odour familiarity, and 13% power to detect the observed, very small effect size for age.

### Participants

In total, 394 individuals (49% women and girls) participated in the study. Ages ranged from 3 to 81 years (*M* = 16.78, *SD* = 14.55, *Median* = 10). For the purpose of the analyses, participants were classified into four developmental groups: early childhood (3–7 years, *n* = 98), middle childhood (8–12 years, *n* = 171), adolescence and emerging adulthood (13–24 years, *n* = 30), and adulthood (25–81 years, *n* = 95; Del Giudice, 2014; Sameroff & Haith, 1996; Sawyer et al., 2018). Detailed characteristics per group and culture are reported in the Supplementary Materials (SM). We included children as young as three years of age to capture the earliest developmental stage at which verbal instructions could be followed and odour identification reliably tested (Cameron & Doty, 2013; Dalton et al., 2013; Dżaman et al., 2013; but see Cavazzana et al., 2017), thereby maximising the span of cultural exposure examined.

The sample comprised 102 Hadza (45% women and girls), 95 Pakistanis (54% women and girls), 84 Dani (46% women and girls), and 113 Scots (51% women and girls). Hadza participants were hunter-gatherers living in the Tanzanian savannah near Lake Eyasi (Marlowe, 2010). Dani were horticulturalists from the New Guinea highlands (Koch, 1974), residing in the Baliem Valley around the town of Wamena (in the Indonesian province of Papua). Both groups lead lifestyles that differ substantially from those typical of Western cultures; for instance, some members still rely on food-gathering or hunting, and often have limited access to electricity and running water. Pakistani participants were recruited from peri-urban areas of Lahore (representing a non-Western, industrialised urban population), while Scottish participants were recruited in Stirling (representing a Western, industrialised urban population). We acknowledge that while the Hadza and Dani represent distinct small-scale societies with relatively high internal homogeneity, the samples from Lahore and Stirling represent broader urban populations where cultural norms may be less uniform.

All participants provided informed consent prior to participation. Individuals below 18 years old participated in the study with a legal guardian or parent present throughout the procedure. Participation was voluntary. Participants were compensated either directly or indirectly (with the exception of participants from Pakistan, who did not receive compensation). In the case of Hadza children from a boarding school, a donation was made to the school to support the development of its infrastructure). In Stirling, children received a colourful sticker to wear on their clothing. In Papua, parents were compensated, which enabled them to purchase food for their families, including their children.

### Materials

Odours were presented to participants using custom-made stimuli resembling those from the Sniffin’ Sticks test (Hummel et al., 2007). In total, we used 11 odour samples: banana (5% solution of isoamyl acetate in propylene glycol), peach (Takasago, DG FLO 432 L), cinnamon (5% solution of cinnamaldehyde in propylene glycol), jasmine (Takasago, DG FLO 770 G), strawberry (Takasago, DG FLO 791 B), fish (2% solution of Squid Brand Fish Sauce [Nam Pia] in propylene glycol), garlic (Destilla GmbH, Knoblauch Gewuerz Aroma), thyme (Frey&Lau, Thymianoel EuAB P0123774), butter (Frey&Lau, Nat. Aroma, Typ cremige Butter, P0620830), ginger (Frey&Lau, Ingweroel, S0100054), and n-butanol (5% solution of n-Butanol in propylene glycol).

Each participant reported their age (in years) and gender, before rating the pleasantness of each odour (*Do you like this smell?* with five possible responses: 1 = *I really don’t like it,* 2 = *I don’t like it,* 3 = *not sure,* 4 = *I like it,* 5 = *I really like it*). Each pleasantness rating option was accompanied by a corresponding smiley emoji (see SM). We acknowledge that the verbal label “*not sure*” may imply uncertainty rather than hedonic neutrality, which poses a limitation to the current study. However, the accompanying neutral face emoji should have helped anchor the mid-point as “*neither like nor dislike*,” minimising potential ambiguity. To simplify the response format for the Hadza participants, who are generally unaccustomed to Likert-type scales, a 3-point scale was used (1 = *I don’t like it*, 2 = *not sure*, 3 = *I like it*). Participants were also asked whether they were familiar with each odour (*Do you know it?* [the smell]), with responses on a 3-point scale (1 = *no*, 2 = *not sure*, 3 = *yes*).

Importantly, we used a unified testing procedure (i.e. a rating scale anchored by emojis) across all age groups. We acknowledge that some prior studies have employed different methods for young children (e.g. forced-choice ranking; Schmidt & Beauchamp, 1988). However, using a consistent measure was essential in the present study to enable direct comparisons of both variability and mean levels across different age groups, without the confounding effects of differing task structures.

### Procedure

The study was conducted in the local language spoken by participants at each study site, except among Hadza, for whom Swahili (a language commonly understood within this group) was used. For Hadza children from the boarding school, a local Hadza assistant (a teacher) was present to clarify any questions in Hadzane. Each participant (or a legal guardian/parent of young children) was first asked to provide informed consent to participate. Testing was conducted individually, with a legal guardian or parent present for all child participants.

After obtaining consent, we ensured that each participant understood the evaluation task and had time to familiarise themselves with the way that odour stimuli would be presented. Participants then rated the pleasantness and familiarity of the 11 odours. A researcher demonstrated how to present one odour to children and their parents by removing the cap from the Sniffin’ stick, holding the end in front of the child’s nose, and encouraging them to evaluate it. They were allowed to assess the odour for as long as they wished, and then asked to provide the rating. For younger children, either a parent or a researcher continued to do the same for all the stimuli. Older children and adults self-administered the stimuli. In all cases, the order was semi-random, with items completed in an arbitrary order until all stimuli had been assessed.

### Statistical analysis

To account for differences in response scales (3-point versus 5-point), odour pleasantness ratings were converted into per cent of maximum possible (POMP) scores (Cohen et al., 1999). The POMP transformation adjusts for differences in scale range while preserving underlying variation in responses (Mellenbergh, 2019). Two variables were then computed: age factor (with four groups, including early childhood with participants between 3 and 7 years old, middle childhood with participants between 8 and 12 years old, emerging adulthood with participants between 13 and 24 years old, and adulthood group with participants older than 25 years old), and a pleasantness variable (with the three most negatively rated odours across the total sample: Fish, Garlic, Butter as the reference category versus the three most positively rated odours across the total sample: Strawberry, Peach, and Jasmine). Except for post hoc analyses, for which a Tukey correction for multiple testing was applied, statistical inferences were conducted at an alpha level of.05.

To test the hypotheses, a series of linear mixed-effects models was fitted using maximum likelihood estimation. In these models, the dependent variable was food odour pleasantness (POMP scores). A random intercept for participants was included to account for individual differences in overall odour pleasantness ratings. Depending on the model, predictors included culture (Dani, Hadza, Pakistani, and Scots), odour type (Banana, Peach, Cinnamon, Jasmine, Strawberry, Fish, Garlic, Thyme, Butter, Ginger, and Butanol), a binary-coded pleasantness variable (with the three most negatively rated food odours: Fish, Garlic, and Butter as the reference category versus the three most positively rated odours: Jasmine, Strawberry, Peach), the continuous age variable (age in years), odour familiarity, and interactions.

To test H1 (that *members of four distinct cultures rate odour pleasantness similarly*), heteroscedastic residual variances across odour categories were modelled using a variance function structure in the first set of models (for each of the four cultures). The overall residual standard deviation (σ) was obtained from the model’s sigma estimate. For each odour, the absolute residual standard deviation was then computed by multiplying the odour-specific weight by the reference residual standard deviation. This procedure yielded the residual variability (σ) around each odour’s fitted mean, along with 95% confidence intervals, allowing for a direct comparison of heterogeneity in pleasantness ratings across cultures. The proportion of variance explained by the fixed effect of culture was also examined. Furthermore, Spearman’s rank correlation coefficients were computed from the mean pleasantness ratings of the 11 odours for each pair of cultures.

To test H2 (that *odour pleasantness ratings are more consistent (less variable) in younger than in older age groups*), the same modelling strategy was applied to the subsamples corresponding to each of the four age groups. In addition, the amount of variance explained by the fixed effect of the continuous age variable was examined.

To test H3 (that *the most unpleasant odours are rated more similarly across cultures than the most pleasant odours*), the same modelling strategy was applied to the full sample, with the binary-coded valence variable included (with the three most negatively rated odours across the total sample: Fish, Garlic, and Butter as the reference category, and the three most positively rated odours across the total sample: Jasmine, Strawberry, and Peach as the comparison group). Here, too, the variance explained by the fixed effect of odour pleasantness was assessed.

To test H4 (that *familiarity is strongly positively related to odour pleasantness ratings*), odour familiarity was included as a predictor, allowing for the examination of its association with pleasantness ratings and the amount of variance it explained.

Finally, a model including odour familiarity and the interactions between culture, odour type, and continuous age variable was fitted, followed by a model without interactions. Post-hoc analyses were then conducted to compare pairs of odours and cultures, using estimated marginal means (EMMs) derived from the fitted model, with Tukey-adjusted *p* values to control for Type I error rates associated with multiple comparisons.

## Results

**Fig. 1:**
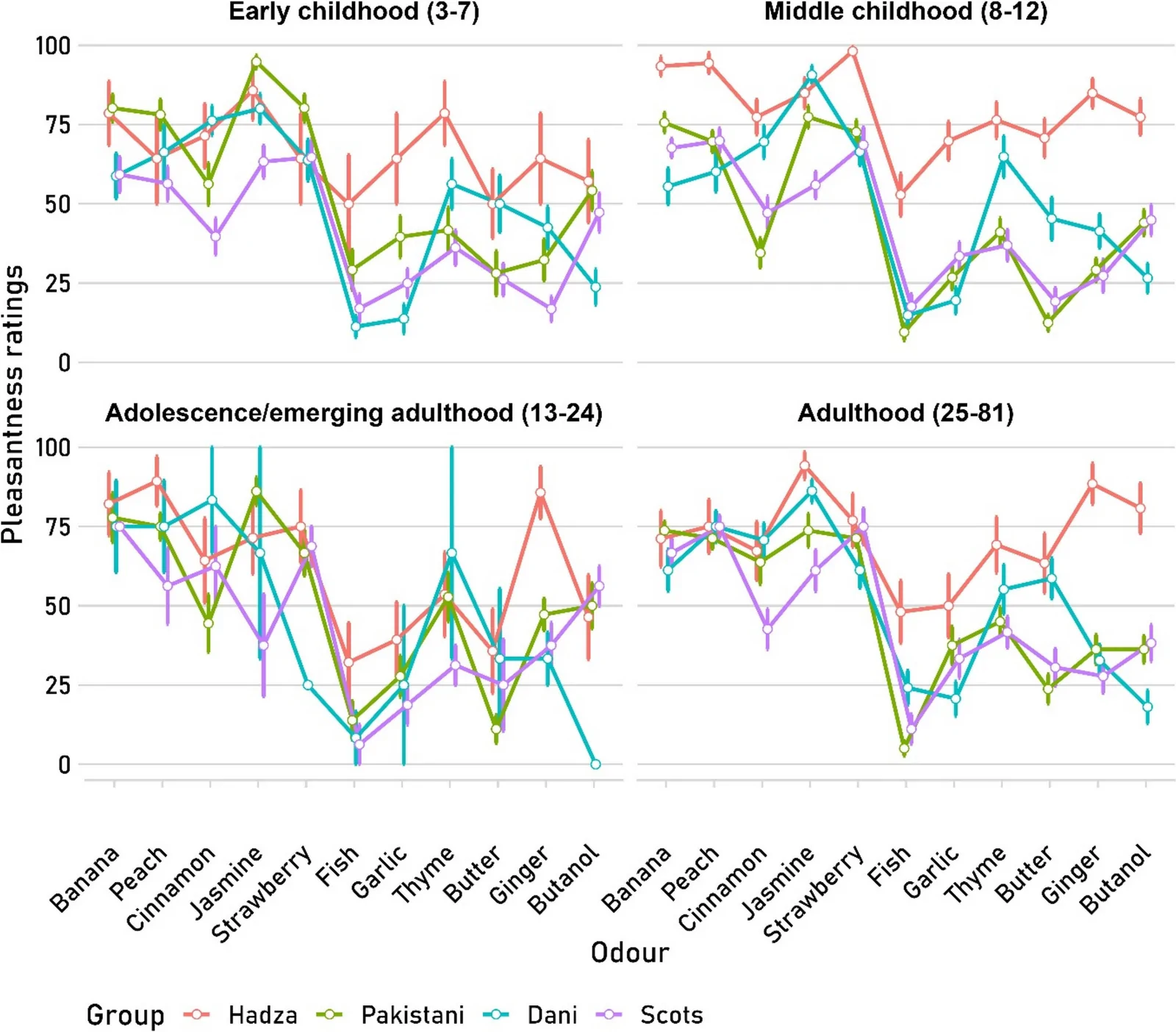
Odour pleasantness ratings (POMP scores) across the four cultures and four age groups. Error bars represent standard errors. (Colour figure online)

**Fig. 2:**
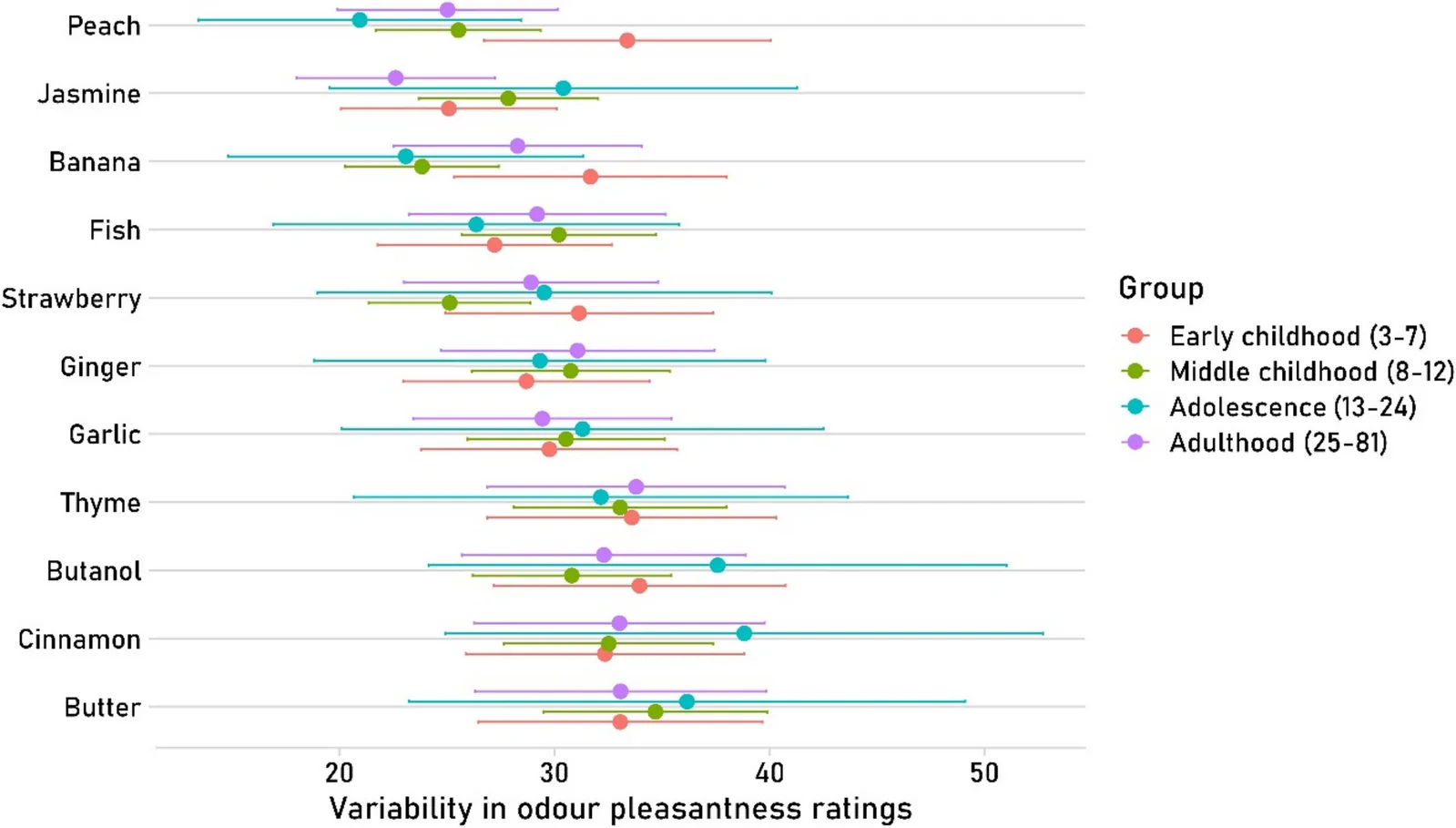
Variability in odour pleasantness ratings across four age groups of participants (the lower the score, the higher the similarity in odour pleasantness ratings; conversely, the higher the score, the higher the variability in odour pleasantness ratings). Error bars represent 95% confidence intervals. (Colour figure online)

A summary of the hypotheses and results is presented in Table 1. Average odour pleasantness scores across the four cultures, as well as for the four different age group subsamples, are presented in Table S1 in the SM, with a visual representation shown in Figure 1. Residual variability across odours, cultures, and age groups is presented in Table S2 (for graphical representations, see Fig. 2). Table 2 reports simplified results of the model with the interaction between culture, type of odour, and age and odour familiarity. Table S3 shows detailed results of this model, Table S4 shows the results without interactions, and Table S5 shows results of post-hoc analyses with Tukey correction, comparing variability between pairs of cultures and odours.

**Table 2:** A summary of results of the multilevel model with odour pleasantness ratings regressed on culture, participants’ age (continuous variable; in years), type of odour (and their interaction), and odour familiarity (responses nested within participants)

Fixed effects	*F*	*p*	AIC	BIC	*df*	ICC	Pseudo *r*^2^ (fixed)	Pseudo *r*^2^ (total)
Culture	25.548	<.001***	41137.1	41715.7	4172	.156	.382	.479
Type of odour	55.109	<.001***	**Random effects**	**Variance**	***SD***			
Age	1.470	.226	Participant	145.5	12.06			
Odour familiarity	235.869	<.001***	Residual	787	28.05			
Culture × Age	1.251	.291						
Type of odour × Age	0.822	.607						
Culture × Type of odour	4.667	<.001***						
Culture × Type of odour × Age	1.630	.017*						

*p <.05, **p <.01, ***p <.001. AIC = Akaike information criterion; BIC = Bayesian information criterion; ICC = intraclass correlation coefficient

### Is there cross-cultural similarity in ratings of odour pleasantness?

Analyses of the heteroscedastic residual variances revealed that for four odours (Jasmine, Ginger, Cinnamon, and Thyme), the degree of within-culture consensus (i.e. variability in ratings) was similar across the four cultures. However, for the remaining 64% of odours, significant differences in rating variability were observed, indicating that consensus on these odours varied across the four groups. Detailed analyses of the variability in odour ratings across cultures are presented in the SM. The full multilevel model including all fixed effects and interactions indicated significant between-culture differences in odour pleasantness ratings, both as a main effect, *F*_(3, 396)_ = 25.548, *p* <.001) and in interaction with odour type, *F*_(30, 3874.9)_ = 4.667, *p* <.001 (for full results, see Table S3 in the SM). At the same time, Spearman’s rank correlations showed relatively high agreement in odour pleasantness ratings across cultures (mean ρ =.79; see Table S6 for detailed results). However, inspection of mean pleasantness ratings revealed specific cultural differences. For instance, while Fish was the lowest-ranked odour across all cultures, Hadza participants rated it relatively less negatively (closer to the mid-point of the response range; *M* = 48.5, *SD* = 47.91), while Pakistani participants rated it relatively more negatively (closer to the lower end of the response range; *M* = 13.95, *SD* = 22.10). These results indicate high cross-cultural agreement in preference hierarchy, but significant variability in both absolute ratings and within-culture consensus, providing mixed support for H1 (that *members of four distinct cultures similarly rate odour pleasantness*).

### Are pleasantness ratings more similar among younger than older individuals?

The degree of within-culture consensus (i.e. variability in ratings) did not differ substantially between younger children (aged 3–7), older children (aged 8–12), adolescents (aged 13–24), and adults (aged 25–81) for any of the odours (see Table S2 in the SM for details). As illustrated in Fig. 2, the confidence intervals for the variability estimates (representing the dispersion of scores) of all age groups overlapped substantially across all 11 odours. This pattern held consistently regardless of the odour’s valence, from positively rated scents like Jasmine and Peach to negatively rated ones like Fish and Garlic.

**Fig. 3:**
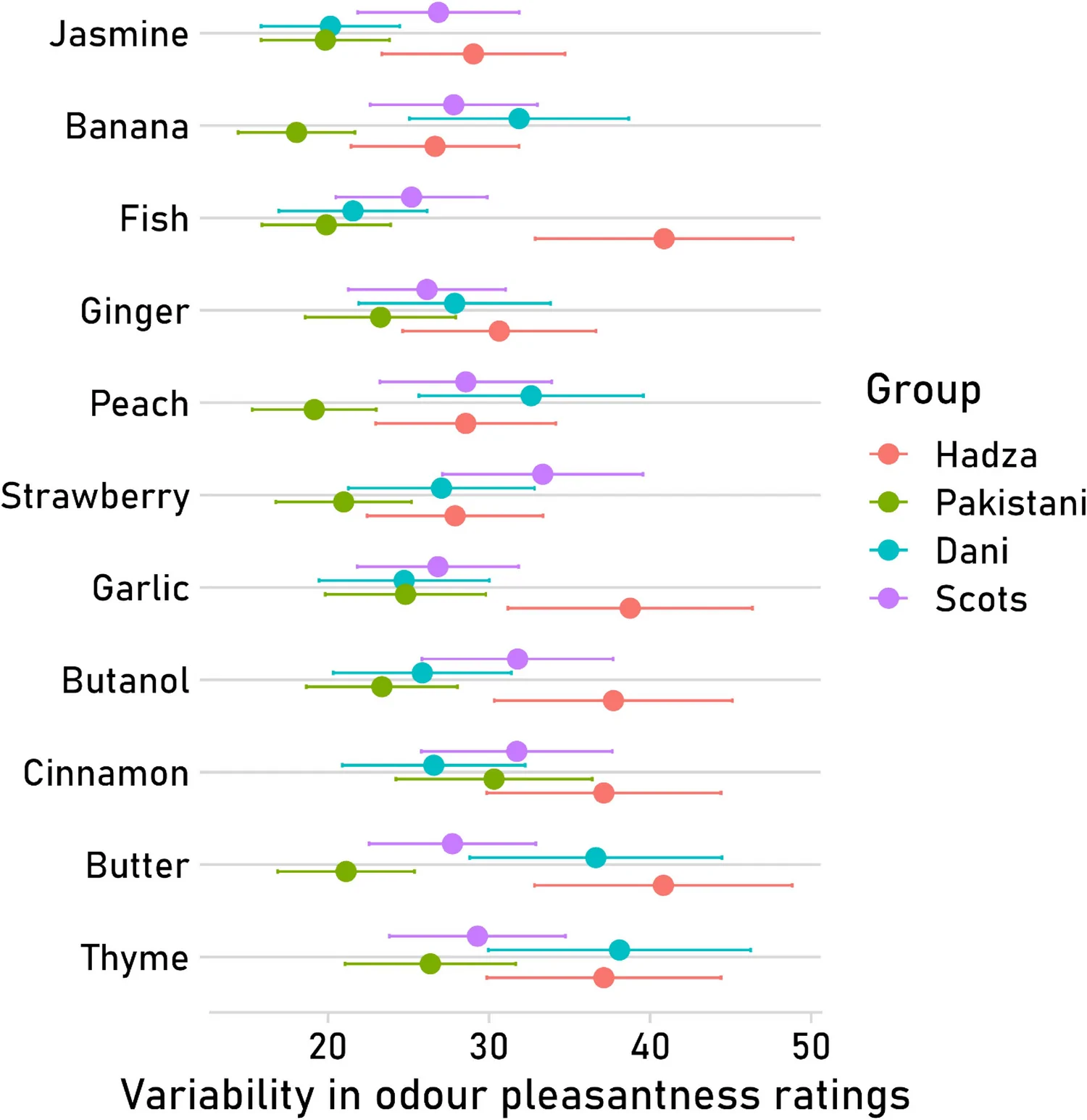
Variability in odour pleasantness ratings across four cultures (the lower the score, the higher the similarity in odour pleasantness ratings; conversely, the higher the score, the higher the variability in odour pleasantness ratings). Error bars represent 95% confidence intervals. (Colour figure online)

The age variable explained less than .09% of the variance. The multilevel model including all fixed effects and interactions did not reveal any significant differences in pleasantness ratings across participants’ age in terms of the main effect, *F*_(1, 390.8)_ = 1.470, *p* =.226, the interaction with culture, *F*_(3, 389.6)_ = 1.251, *p* =.291, or the interaction with odour type, *F*_(10, 3874.5)_ = 0.822, *p* =.607. Only the three-way interaction with culture and odour type was significant, *F*_(30, 3874.6)_ = 1.630, *p* =.017. However, this effect was probably driven by unusually high odour pleasantness ratings in Hadza children and adults (see Fig. 3). To conduct a more fine-grained analysis, a subsample of participants aged 3–15 was further divided into six 2-year age groups (3–4, 5–6, 7–8, 9–10, 11–12, and 13–15). Analysis of variability in pleasantness ratings showed significant differences only between the 3–4-year-olds (σ = 43.279) and the 9–10-year-olds (σ = 34.121), and between the 3–4-year-olds and the 13–15-year-olds (σ = 29.529; see Fig. S1 and Table S7 for details). Finally, to address concerns related to the challenges of testing children younger than 5 years (Cameron et al., 2024) and a decline in olfactory function with advancing age (Doty et al., 1984; Murphy et al., 2002), we conducted a sensitivity analysis excluding data from children younger than 5 years and adults older than 60 years. The results remained virtually unchanged (see Tables S8–S9 in the SM). Taken together, these findings do not support H2 (that *odour pleasantness ratings are more consistent [less variable] in younger than in older age groups*).

### Are the most unpleasant odours rated more similarly than the most pleasant odours?

Variability in pleasantness ratings was lower for the three most pleasant odours (rated across the full sample: Jasmine, Strawberry, and Peach, σ = 27.001) than for the three least pleasant odours (again rated across the full sample: Fish, Garlic, and Butter, σ = 33.097; see Fig. S2). The binary-coded valence variable explained 27.2% of the variance. As expected, the multilevel model including all main effects and interactions with the binary coded valence variable indicated significant differences between the most and least pleasant odours, *F*_(1, 2152.83)_ = 62.894, *p* <.001. Importantly, the interaction with culture was also significant, *F*_(3, 1956.31)_ = 15.625, *p* ≤.001, indicating that the difference in variability between pleasant and unpleasant odours differed across cultures. These results counter H3 (that *the most unpleasant odours are rated more similarly across cultures than the most pleasant odours*), as the opposite pattern was observed. The full results of the multilevel model are presented in Table S10.

Although the clustering of the three most and least pleasant odours (based on the full sample means) was relatively high (e.g. Fish was rated as the least pleasant odour in all cultures), some differences in rank order occurred. Therefore, as a robustness check, we compared the single least pleasant odour across all cultures (Fish) with Jasmine, which was the single most pleasant odour for two cultures (Pakistani and Dani), the third most pleasant for the Hadza, and the fourth most pleasant for the Scots. The results of this specific comparison were consistent with the main analysis. Specifically, variability in pleasantness ratings was lower for Jasmine (σ = 24.337), which consistently ranked among the most pleasant odours, than for Fish (σ = 30.835), which was consistently rated as the least pleasant odour.

### Is familiarity related to odour pleasantness ratings?

Odour familiarity was strongly positively associated with pleasantness ratings (β = 0.396, *SE* = 0.104, *p* <.001, 95% CI [0.192, 0.600]), providing support for H4 (that *familiarity is strongly positively related to odour pleasantness ratings*). Familiarity explained 13.5% of the variance in pleasantness ratings. Furthermore, results from the multilevel model with interactions revealed that the strength of the familiarity–pleasantness association varied across cultures, *F*_(3, 4170.1)_ = 12.238, *p* <.001, and across specific odours, *F*_(10, 4054.3)_ = 4.569, *p* <.001. The full results are presented in Table S11 in the SM.

## Discussion

This study aimed to explore the extent to which odour preferences reflect shared sensory predispositions and how much they are shaped by ecological and cultural environments. We tested four specific hypotheses derived from previous literature (e.g. Arshamian et al., 2022; Oleszkiewicz et al., 2022; Schaal et al., 1996; Schmidt & Beauchamp, 1988; Sorokowski et al., 2024), using a sample of children and adults from four distinct cultures (Hadza, Pakistani, Dani, and Scots).

### Cross-cultural (dis)agreement in odour pleasantness ratings (H1)

We found mixed support for our first hypothesis, which predicted agreement in odour pleasantness ratings across cultures. On one hand, the degree of within-culture consensus in pleasantness ratings was similar for four out of 11 (36%) odours, and the overall order of odour pleasantness was relatively consistent across cultures, as indicated by a high Spearman’s rank correlation. On the other hand, the degree of within-culture consensus varied across cultures for the remaining seven out of 11 odours (64%), and multilevel analyses further revealed significant differences in mean odour pleasantness ratings between cultures. Taken together, our findings are consistent with prior research pointing to both a universal consensus in odour evaluations (Arshamian et al., 2022; Oleszkiewicz et al., 2022) and the predominant role of ecological and cultural influences in shaping olfactory perception (Sorokowski et al., 2024).

Some disagreement in odour pleasantness ratings may stem from factors such as dietary traditions and the ecological availability of certain foods. For instance, the greatest variability (i.e. lack of consensus) in the liking of fish odour was observed among the Hadza people. This may reflect heterogeneous exposure to this odour. Some individuals lived closer to Lake Eyasi and perhaps more frequently consumed fish than others, leading to variability in Fish pleasantness evaluations. In a similar vein, cultural differences in dietary consistency may explain differences in consensus regarding butter. Of the four cultures, Pakistanis are most likely to frequently consume butter/ghee (Pervaiz et al., 2022); they also exhibited the lowest rating variability, suggesting that consistent dietary exposure may translate to more uniform olfactory opinions. These patterns for variability were not fully reflected in average familiarity ratings, thus warranting further investigation into how the frequency of eating specific foods impacts odour perception consensus.

### Agreement in odour pleasantness ratings across age groups (H2)

We did not find support for our second hypothesis, which predicted larger agreement in odour pleasantness ratings in younger than older age groups: variability in odour liking was similar across the four age groups, including younger children (aged 3–7), older children (aged 8–12), adolescents (aged 13–24), and adults (aged 25–81). Given the lack of evidence for H1 (concerning agreement about particular odours), this finding suggests that familial socialisation likely aligns children’s food‐related preferences with those of their parents through shared meals and exposure. This finding is also consistent with the work of Mennella and colleagues, which suggests that chemosensory preferences are shaped very early in life, even prenatally, through the transfer of flavours from the maternal diet into amniotic fluid and breast milk (Mennella & Beauchamp, 1991; Mennella et al., 2001). Such early exposure lays the foundation for culturally specific odour and flavour preferences, which are further reinforced by dietary practices during childhood. However, it is worth noting that we could not make direct comparisons between children and their parents, as we lacked such information.

General agreement in odour pleasantness ratings between participants at different age raises another question: at what age do odour preferences emerge? Our sample consisted of younger children aged 3 to 7 and older children aged 8 to 12, with a mean of 8.27 and a standard deviation of 2.24. Considering that detailed analyses with children categorised into six age groups suggested a pattern of growing uniformity in odour pleasantness ratings, it seems that odour preferences could be shaped gradually across childhood. Such conclusions are supported by prior research showing that children’s hedonic responses to odours start to resemble those of adults from around age seven (Engen, 1974). However, future longitudinal studies are needed to shed more light on how cultural transmission in odour liking occurs as children develop.

### Universality of unpleasantness versus pleasantness (H3)

Our third hypothesis, which focused on the asymmetry in the universality of unpleasant and pleasant odours, was also not supported. We found lower variability in pleasantness ratings of the most pleasant odours than for the most unpleasant. These findings challenge evolutionary interpretations of error management theory (Haselton & Buss, 2000; Oaten et al., 2009), which predict that humans should have evolved greater sensitivity and bias towards detecting potentially dangerous stimuli over preferences towards pleasant and safe stimuli. Interestingly, some prior research has found support specifically for the prediction that unpleasant odours are evaluated more consistently across individuals and cultures than pleasant ones (for a review, see Schaal et al., 2004).

Our results suggest that consensus regarding odour pleasantness may be stronger for more pleasant food-related odours, particularly those associated with sweetness and ripe fruits, such as Jasmine, Peach, and Strawberry, compared with unpleasant food-related odours, such as Fish, Garlic, and Butter. However, the unpleasant food odours used in this study may not have been sufficiently unpleasant to evoke strong adverse reactions, as odours such as faeces, vomit, or spoiled food would (Schienle & Schöpf, 2017; Stevenson, 2010; Wisman & Shrira, 2015). Therefore, more studies are needed to evaluate whether unpleasant odours signalling stronger pathogenic threat are associated with lower variability (i.e. higher consensus) in pleasantness ratings than pleasant odours.

### Familiarity and odour pleasantness (H4)

Finally, we did find support for the fourth hypothesis (that familiarity is positively related to odour pleasantness ratings). This finding is consistent with the “mere exposure effect”, well-documented in prior research on various objects, including smells (e.g. Oleszkiewicz et al., 2022; Rabin & Cain, 1984; Sorokowski et al., 2024). It reinforces the fundamental principle that repeated, non-negative exposure to an odour, typically through dietary habits and cultural practices, increases liking for it. This mechanism may also partly account for the observed differences regarding H1; perhaps participants from all four cultures prefer the odours of the foods they know and regularly consume.

Beyond familiarity, two further factors may have contributed to the observed results, including odour context and perceived intensity. The semantic frame or verbal label applied to an odorant can substantially alter its perceived pleasantness, even when the olfactory stimulus itself remains physically unchanged (Araujo et al., 2005; Herz & von Clef, 2001). In the present study, no verbal labels were provided to participants before rating each odour. Each participant, thus, may have applied their own associations to the odours, and these associations likely differed considerably across cultures. For instance, the relative pleasantness of fish odour among Hadza participants compared with Pakistani participants may reflect not only differential familiarity, but also divergent cultural meanings tied to fish as a food source. Contextual associations of this kind could therefore partly account for the cross-cultural variability in absolute pleasantness ratings observed here, over and above the contribution of familiarity per se. Future studies may thus explore factors linked to odour liking, including odour intensity, context, and dietary habits. In sum, while familiarity was a robust predictor of pleasantness, contextual and semantic factors, such as cultural meanings attached to specific odours, may also contribute to cross-cultural differences in pleasantness ratings, suggesting that odour evaluation reflects more than sensory exposure alone.

## Limitations

An important limitation of our study is the lack of detailed data on participants’ dietary habits. As nearly all odorants used were food-related, quantitative data on dietary exposure would have provided valuable context. Although we assessed perceived odour familiarity as a psychological proxy for prior exposure, we did not measure the actual consumption frequency of specific odour-producing foods. This limitation is largely attributable to the inclusion of very young children and participants from non-industrialised populations, for whom collecting standardised, reliable dietary histories is methodologically challenging. Furthermore, children’s diets may differ qualitatively from those of adults, and the extent of this difference may vary across cultures. Additionally, olfactory exposure could stem not only from one’s own diet but also from family eating practices, adding another interpretative difficulty to the diet-control idea. The lack of specific consumption data prevents us from quantifying the extent to which these developmental dietary nuances influenced the results. Future studies would nevertheless benefit from integrating hedonic ratings with detailed dietary history to better disentangle how specific ingestion frequencies shape olfactory preferences (Aldridge et al., 2009). We also caution that interpretations regarding specific dietary intakes regarding fish in Hadza and butter in Pakistanis are speculative, relying on general ethnographic descriptions and our observations during field studies rather than direct measures of individual consumption.

Second, we did not assess odour identification performance or the linguistic availability of odour labels in the local languages. Prior to the main data collection, a small pilot study was conducted in each of the studied groups to verify that participants were able to engage with the odour stimuli and understand the rating procedure. However, this pilot study did not include a formal assessment of odour identification accuracy. We acknowledge that preferences do not exist in a cognitive vacuum. Without identifying the odour object (e.g. recognising the smell as “Butter”), participants may rely on sensory features rather than semantic associations. Consequently, it is possible that the Fish and Butter (and other) odours were not perceived as representative of their respective food sources, or that participants (especially in the Hadza and Dani groups) did not possess specific lexical terms to describe them. If participants failed to identify the source object, their pleasantness and familiarity ratings might differ from those of participants who successfully labelled the odour, potentially explaining discrepancies between assumed dietary habits and observed ratings. This limitation also tempers the strength of our conclusions regarding H4. While the positive familiarity–pleasantness association was robust, the familiarity ratings reflect subjective recognition responses rather than verified exposure histories or confirmed odour identification. Furthermore, the selection of stimuli imposes constraints on generalizability. The applied set of odorants was relatively small and predominantly food-related. Future studies should include a broader range of odours to provide a more complete picture of human odour preferences.

A related limitation concerns context effects, which represent an important source of variability in hedonic responses to odours. Prior research has provided evidence that a verbal label provided with an odorant can substantially alter its perceived pleasantness, even when the olfactory stimulus itself is held constant (Araujo et al., 2005; Herz & von Clef, 2001). In the present study, no explicit verbal labels were given to participants before they rated each odour. We can assume that intraindividual and cross-cultural variability occurred in that regard. Future studies examining cross-cultural odour preferences should systematically control for, or measure, contextual associations to better isolate the contribution of the olfactory stimulus itself.

Furthermore, we did not explicitly distinguish between pure olfactory stimuli and those with strong trigeminal properties (e.g. irritation). The odour set used in this study included odorants with markedly different trigeminal profiles. Specifically, n-butanol and cinnamon are well-established trigeminal stimulants that can elicit sensations of irritation or warmth in addition to their olfactory qualities (Hummel & Livermore, 2002). Other odorants in the set, such as isoamyl acetate (banana) and peach, are predominantly olfactory in character with only weak trigeminal components. Notably, neither butanol nor cinnamon elicited consistently negative hedonic responses across cultures, despite their trigeminal irritancy, which suggests that even odorants with marked trigeminal properties remain subject to cultural and individual influences on hedonic valence. Because perceived trigeminal activation was not assessed independently in this study, we cannot fully disentangle the contribution of trigeminal versus olfactory processing to participants’ pleasantness ratings.

Although the concentration of each odorant in solution was standardised and reported, perceived odour intensity was not measured. Perceived intensity is known to be related to hedonic valence, with evidence for both linear and nonlinear (inverted-U) functions depending on the odorant (Distel et al., 1999). Importantly, perceived intensity for a given concentration may vary substantially between individuals, and systematic cross-cultural differences in olfactory sensitivity could have contributed to some of the observed variability in pleasantness ratings. Future studies would benefit from including intensity ratings alongside pleasantness and familiarity assessments. Additionally, the cross-sectional nature of the study limits our ability to draw causal inferences regarding the developmental trajectory of odour preferences. While we compared different age groups, only longitudinal designs can definitively track how cultural transmission shapes olfactory preference within individuals over time.

A further limitation concerns the potential influence of developmental and age-related changes in olfactory function. Hormonal changes during puberty have been found to affect olfactory sensitivity and hedonic responses to odours (Doty & Cameron, 2009). Our classification of participants into four broad age groups did not account for individual variation in pubertal onset. It is therefore possible that some older children in our sample were already experiencing puberty-related changes in olfactory perception, which could have introduced some heterogeneity within this group. In contrast, at the other end of the age continuum, olfactory function has been also found to decline substantially with advancing age (Doty et al., 1984; Murphy et al., 2002), meaning that some older adult participants may have had diminished olfactory abilities relative to younger adults. Given that only three participants were above 60 years of age, we do not expect this to have meaningfully affected the overall findings, and this was further confirmed with our sensitivity analyses, in which we excluded all data from participants below 5 and above 60 years old. Nevertheless, future cross-cultural studies spanning a wide age range should carefully account for pubertal stage and age-related olfactory changes.

While our study provides evidence that the youngest children (3–4 years) exhibited significantly greater rating variability than older children (9–10 years), the validity and reliability of odour ratings in children under 5 warrants specific consideration. Prior research has raised concerns about the reliability of odour-related tasks in children younger than 5 years of age (Cavazzana et al., 2017), and the greater variability in the youngest group may partly reflect cognitive difficulties in using a rating scale, or less stable hedonic representations at this age, rather than differences in odour preferences (Cameron et al., 2024). Prior to the main data collection, a brief pilot study was conducted to confirm that all participants, including the youngest children, could understand the procedure and engage meaningfully with the stimuli. However, this pilot did not include a formal assessment of odour identification performance. To evaluate robustness, we re-ran the main analyses excluding children younger than five (*n* = 10) and adults older than 60 (*n* = 3). The results remained virtually unchanged (see Tables S8–S9 in the SM), suggesting that the inclusion of these age groups did not meaningfully affect the overall conclusions. This is also consistent with findings by Schmidt and Beauchamp (1988), who reported adult-like odour preferences in three-year-olds, though differences in methodology (forced-choice ranking versus rating scales) may partly account for discrepancies across studies.

Finally, due to logistical and funding constraints, we were unable to collect representative samples from each culture, especially the hard-to-reach ones (i.e., Hadza and Dani). At the same time, although our study focused on cross-cultural comparisons, it is important to acknowledge the substantial role of individual differences within cultures. Preference variability between individuals often exceeds variability between groups (Wysocki et al., 1991). Our data reflect this, as the random effect for participants accounted for a meaningful portion of the variance. These individual differences likely stem from a combination of genetic variation in olfactory receptors (Mainland et al., 2014) and idiosyncratic personal histories (Herz, 2016). This interpretation aligns with recent work by Arshamian et al. (2022), who demonstrated that while cultural background explains some variance in odour pleasantness, a substantial proportion is driven by individual molecular and experiential factors. Thus, personal preference is a powerful variable shaping hedonic responses alongside broader cultural norms.

## Conclusions

Our results, involving children and adults from four ecologically distinct cultures, address the ongoing debate about the extent to which odour perception is universal or culturally shaped. Although some convergence was observed, particularly for highly familiar and positively valenced food odours, cultural and ecological factors also appear to play a meaningful role in shaping odour preferences. Odour preference variability did not differ substantially across the four age groups studied, suggesting that cultural influences may emerge relatively early in life. Contrary to some evolutionary predictions, the most pleasant odours were rated more consistently than unpleasant ones, suggesting that positive hedonic responses may be more conserved than previously thought. Finally, subjectively reported familiarity emerged as a robust predictor of pleasantness, highlighting the role of dietary exposure and learning in olfactory evaluation. Together, these results contribute to a growing body of work that illustrates the dynamic interplay between biology, experience, and culture in shaping human olfactory perception.

## Supplementary Information

Below is the link to the electronic supplementary material.

Supplementary file1 (DOCX 199 KB)

## Data Availability

All primary data and materials are publicly available (https://osf.io/54dyr).

## Code availability

The analysis script is publicly available (https://osf.io/54dyr).
