# Supplementary material for: Odour preferences of children and adults across four diverse cultures in Africa, Asia, Europe, and Oceania
Source: Psychon Bull Rev. 2026 Oct 26;33(8):267. doi: 10.3758/s13423-026-02984-7 (PMC13710644; doi:10.3758/s13423-026-02984-7)
Supplement: Supplementary file 1 [file 13423_2026_2984_MOESM1_ESM.docx]

# Supplementary Materials

There were 43 (43.9%) women in the group 3-7 (age *M* = 5.82, *SD* = 1.02); 83 (48.5%) women in the group 8-12 (age *M* = 9.68, *SD* = 1.37); 15 (50.0%) women in the group 13-24 (age *M* = 18.03, *SD* = 3.84); 47 (49.5%) women in the group 25-81 (age *M* = 40.49, *SD* = 9.47). Average age in Hadza children was 9.43 (*SD* = 1.81), Pakistani 8.21 (*SD* = 2.05), Dani 8.35 (*SD* = 2.01), and Scots 7.51 (*SD* = 2.45). Average age in Hadza adults was 31.74 (*SD* = 11.51), Pakistani 30.45 (*SD* = 11.68), Dani 40.62 (*SD* = 10.76), Scots 39.64 (*SD* = 15.42). There were 60 (59%) children in Hadza, 66 (70%) children in Pakistani, 52 (62%) children in Dani, and 91 (80%) children in Scots.

In general, Hadza rated odours as the most pleasant (*M_(POMP)_* = 74.204, *SD_(POMP)_* = 41.392), followed by Dani (average *M_(POMP)_* = 50.244, *SD_(POMP)_* = 36.827), Pakistani (average *M_(POMP)_* = 48.971, *SD_(POMP)_* = 33.416), and Scots (average *M_(POMP)_* = 43.176, *SD_(POMP)_* = 35.843).

Participants from Pakistan showed the highest agreement in odour pleasantness ratings (average σ = 22.463), followed by Dani (average σ = 28.452), Scots (average σ = 28.655), and Hadza (average σ = 34.107), who exhibited the greatest variability in their ratings, indicating the lowest level of agreement on odour pleasantness. Post-hoc analyses using estimated marginal means (EMMs) of the POMP scores indicated that the Hadza rated the odours as significantly more pleasant than participants from other cultures (EMM = 68.8, SE = 1.53; all pairwise comparisons *p* < .001). Pakistani and Dani participants provided intermediate ratings (EMM = 50.7, SE = 1.57, and EMM = 50.6, SE = 1.64, respectively), both significantly higher than those of Scots (EMM = 44.4, SE = 1.52; Pakistani vs. Scots: *p* = .016; Dani vs. Scots: *p* = .026).

Among the 11 odours, Jasmine exhibited the lowest variability in pleasantness ratings (σ = 26.332), indicating the highest level of agreement among participants. It was followed by Banana (σ = 27.222), Peach (σ = 27.444), Strawberry (σ = 28.140), Fish (σ = 28.875), Garlic (σ = 30.276), Ginger (σ = 31.270), Butanol (σ = 32.680), Cinnamon (σ = 33.116), Thyme (σ = 33.341), and Butter (σ = 34.498), which showed the highest variability. Post-hoc analyses revealed that Jasmine (EMM = 74.9, SE = 1.65), Peach (EMM = 70.8, SE = 1.64), and Strawberry (EMM = 70.2, SE = 1.65) were rated as the most pleasant odours and did not significantly differ from each other (all pairwise *p* > .48). Banana (EMM = 67.1, SE = 1.66) was rated slightly less pleasant, though not significantly different from Peach (p = .82) or Strawberry (p = .94). However, Banana was rated significantly more pleasant than Cinnamon and all lower-ranked odours (all *p* < .01). Cinnamon (EMM = 58.1, SE = 1.64) occupied a mid-range position, rated significantly below Banana but above Thyme (EMM = 53.1, SE = 1.64; p = .04). Pleasantness ratings declined further across Butanol (EMM = 49.9, SE = 1.65), Ginger (EMM = 46.0, SE = 1.64), Butter (EMM = 40.4, SE = 1.64), and Garlic (EMM = 36.2, SE = 1.64), with each pairwise comparison yielding significant differences (all *p* < .01). Fish was rated as the least pleasant odour (EMM = 23.0, SE = 1.64), with all pairwise comparisons against higher-rated odours reaching significance (*p* < .001). Detailed results of the post-hoc analyses are presented in Table S5 in the SM.

For three odours (Strawberry, Butanol, and Butter), one culture (Pakistani) showed significantly lower variability compared to another: lower than Scots for Strawberry, lower than Hadza for Butanol, and lower than both Dani and Scots for Butter. For three odours (Banana, Peach, and Garlic), two cultures differed in variability: Pakistani participants showed less variability than both Scots and Dani for Banana and Peach, while Hadza showed greater variability than both Pakistani and Dani participants for Garlic. For Fish, the Hadza exhibited significantly more variability in pleasantness ratings than participants from the other three cultures. The fixed effect of culture explained 10% of the variance in pleasantness ratings.

Survey

| **Smell** | **Do you like this smell?** | | | | |  | **Do you know it?** | | | **Guess what it is…** |
| --- | --- | --- | --- | --- | --- | --- | --- | --- | --- | --- |
|  | I really don’t like it   | I don’t like it   | Not sure   | I like it   | I really like it   |  | No | Not sure | Yes | *Leave blank if you don’t know it* |
| **Example** | **x** |  |  |  |  |  |  |  | **x** | **Poo!** |
| **1** |  |  |  |  |  |  |  |  |  |  |
| **2** |  |  |  |  |  |  |  |  |  |  |
| **3** |  |  |  |  |  |  |  |  |  |  |
| **4** |  |  |  |  |  |  |  |  |  |  |
| **5** |  |  |  |  |  |  |  |  |  |  |
| **6** |  |  |  |  |  |  |  |  |  |  |
| **7** |  |  |  |  |  |  |  |  |  |  |
| **8** |  |  |  |  |  |  |  |  |  |  |
| **9** |  |  |  |  |  |  |  |  |  |  |
| **10** |  |  |  |  |  |  |  |  |  |  |
| **11** |  |  |  |  |  |  |  |  |  |  |

**Table S1**

Average odour pleasantness ratings (POMP scores) in the full sample, four age subgroups, and four cultures (Hadza, Pakistani, Dani, Scots).

|  | **Full sample** | **Early childhood (3-7)** | **Middle childhood (8-12)** | **Adolescence/emerging adulthood (13-24)** | **Adulthood (25-81)** | **Hadza** | **Pakistani** | **Dani** | **Scots** |
| --- | --- | --- | --- | --- | --- | --- | --- | --- | --- |
| **Odour** | **Mean (SD)** | **Mean (SD)** | **Mean (SD)** | **Mean (SD)** | **Mean (SD)** | **Mean (SD)** | **Mean (SD)** | **Mean (SD)** | **Mean (SD)** |
| Banana | 71.42 (30.33) | 65.72 (33.13) | 75.29 (26.98) | 79.17 (28.68) | 67.74 (32.47) | 85 (32.95) | 76.58 (19.23) | 58.93 (32.62) | 64.29 (28.22) |
| Peach | 72.70 (30.68) | 64.29 (35.17) | 75.58 (29.20) | 79.17 (25.50) | 74.19 (28.42) | 86.5 (32.45) | 72.63 (20.33) | 67.26 (33.60) | 64.60 (30.20) |
| Cinnamon | 58.08 (37.42) | 53.61 (36.44) | 57.60 (38.36) | 60.00 (39.72) | 63.04 (35.83) | 72.5 (42.27) | 47.11 (32.59) | 72.02 (27.00) | 43.92 (35.23) |
| Jasmine | 77.18 (29.59) | 76.02 (30.02) | 76.92 (29.88) | 70.83 (37.76) | 80.91 (25.38) | 85.5 (32.79) | 81.84 (21.09) | 85.71 (21.00) | 59.23 (30.88) |
| Strawberry | 73.08 (30.94) | 68.30 (33.37) | 78.38 (28.12) | 66.67 (34.32) | 70.43 (31.05) | 87.0 (31.48) | 73.68 (21.38) | 62.50 (27.85) | 68.02 (35.06) |
| Fish | 23.98 (34.84) | 21.17 (30.32) | 26.02 (36.72) | 20.83 (34.79) | 24.19 (36.01) | 48.5 (47.91) | 13.95 (22.10) | 16.96 (22.35) | 15.93 (26.53) |
| Garlic | 35.64 (36.42) | 29.12 (32.21) | 40.50 (38.55) | 31.67 (34.70) | 34.78 (36.31) | 60.1 (46.28) | 32.37 (27.01) | 18.75 (25.41) | 29.46 (29.30) |
| Thyme | 52.17 (38.29) | 44.64 (37.35) | 55.41 (38.97) | 51.67 (39.90) | 54.30 (36.98) | 71.5 (42.79) | 43.16 (28.35) | 59.52 (38.59) | 37.17 (32.41) |
| Butter | 38.30 (39.58) | 33.16 (36.22) | 38.53 (41.52) | 26.67 (38.24) | 47.04 (38.46) | 62.5 (46.26) | 18.68 (23.62) | 50.60 (37.02) | 24.11 (30.37) |
| Ginger | 44.63 (37.68) | 29.38 (32.28) | 48.25 (39.54) | 62.50 (32.00) | 48.12 (36.53) | 84.5 (33.1) | 33.16 (24.85) | 38.39 (28.37) | 23.44 (29.31) |
| Butanol | 47.31 (37.63) | 44.85 (36.61) | 51.32 (37.50) | 44.17 (38.66) | 43.48 (38.49) | 72.5 (42.86) | 45.53 (25.78) | 22.02 (26.44) | 45.27 (34.46) |

**Table S2**

Residual variability across the smells, cultures, and four age subgroups, including early childhood (3-7), middle childhood (8-12), adolescence/emerging adulthood (13-24), and adulthood (25-81) (the lower the score, the higher the similarity in odour pleasantness ratings; conversely, the higher the score, the higher the variability in odour pleasantness ratings).

| **Odour** | **Variability** | **95%CI** | **Group** | **Odour** | **Variability** | **95%CI** | **Culture** | **Odour** | **Variability** | **95%CI** |
| --- | --- | --- | --- | --- | --- | --- | --- | --- | --- | --- |
| Banana | 27.222 | [21.886,32.557] | Early childhood (3-7) | | | | Hadza |  |  |  |
| Peach | 27.444 | [22.065,32.823] |  | Banana | 31.681 | [25.343,38.018] |  | Banana | 26.645 | [21.422,31.867] |
| Cinnamon | 33.116 | [26.626,39.607] |  | Peach | 33.393 | [26.713,40.073] |  | Peach | 28.552 | [22.956,34.148] |
| Jasmin | 26.322 | [21.163,31.481] |  | Cinnamon | 32.348 | [25.877,38.819] |  | Cinnamon | 37.128 | [29.851,44.405] |
| Strawberry | 28.140 | [22.625,33.656] |  | Jasmin | 25.093 | [20.074,30.113] |  | Jasmin | 29.028 | [23.339,34.718] |
| Fish | 28.875 | [23.216,34.535] |  | Strawberry | 31.142 | [24.912,37.372] |  | Strawberry | 27.889 | [22.423,33.356] |
| Garlic | 30.276 | [24.342,36.210] |  | Fish | 27.216 | [21.772,32.660] |  | Fish | 40.868 | [32.858,48.878] |
| Thyme | 33.341 | [26.806,39.876] |  | Garlic | 29.766 | [23.811,35.720] |  | Garlic | 38.761 | [31.164,46.359] |
| Butter | 34.498 | [27.736,41.260] |  | Thyme | 33.6 | [26.879,40.321] |  | Thyme | 37.128 | [29.851,44.405] |
| Ginger | 31.270 | [25.141,37.399] |  | Butter | 33.06 | [26.446,39.673] |  | Butter | 40.826 | [32.824,48.827] |
| Butanol | 32.690 | [26.283,39.097] |  | Ginger | 28.697 | [22.956,34.438] |  | Ginger | 30.634 | [24.630,36.639] |
|  |  |  |  | Butanol | 33.957 | [27.164,40.750] |  | Butanol | 37.723 | [30.329,45.117] |
|  |  |  | Middle childhood (8-12) | | | | Dani |  |  |  |
|  |  |  |  | Banana | 23.84 | [20.267,27.414] |  | Banana | 31.865 | [25.05,38.679] |
|  |  |  |  | Peach | 25.535 | [21.707,29.362] |  | Peach | 32.612 | [25.638,39.586] |
|  |  |  |  | Cinnamon | 32.527 | [27.651,37.402] |  | Cinnamon | 26.569 | [20.887,32.251] |
|  |  |  |  | Jasmin | 27.853 | [23.678,32.028] |  | Jasmin | 20.152 | [15.842,24.461] |
|  |  |  |  | Strawberry | 25.128 | [21.362,28.894] |  | Strawberry | 27.044 | [21.260,32.827] |
|  |  |  |  | Fish | 30.203 | [25.676,34.730] |  | Fish | 21.55 | [16.941,26.158] |
|  |  |  |  | Garlic | 30.538 | [25.961,35.115] |  | Garlic | 24.728 | [19.440,30.016] |
|  |  |  |  | Thyme | 33.055 | [28.101,38.010] |  | Thyme | 38.099 | [29.952,46.247] |
|  |  |  |  | Butter | 34.7 | [29.499,39.901] |  | Butter | 36.631 | [28.797,44.464] |
|  |  |  |  | Ginger | 30.757 | [26.147,35.367] |  | Ginger | 27.862 | [21.903,33.820] |
|  |  |  |  | Butanol | 23.84 | [20.267,27.414] |  | Butanol | 25.855 | [20.326,31.384] |
|  |  |  | Adolescence/emerging adulthood (13-24) | | | | Pakistani |  |  |  |
|  |  |  |  | Banana | 23.086 | [14.825,31.347] |  | Banana | 18.046 | [14.417,21.675] |
|  |  |  |  | Peach | 20.948 | [13.452,28.445] |  | Peach | 19.144 | [15.294,22.993] |
|  |  |  |  | Cinnamon | 38.832 | [24.936,52.728] |  | Cinnamon | 30.309 | [24.214,36.404] |
|  |  |  |  | Jasmin | 30.413 | [19.530,41.296] |  | Jasmin | 19.834 | [15.846,23.823] |
|  |  |  |  | Strawberry | 29.523 | [18.959,40.088] |  | Strawberry | 20.973 | [16.755,25.190] |
|  |  |  |  | Fish | 26.367 | [16.932,35.802] |  | Fish | 19.891 | [15.891,23.891] |
|  |  |  |  | Garlic | 31.309 | [20.105,42.513] |  | Garlic | 24.816 | [19.826,29.806] |
|  |  |  |  | Thyme | 32.16 | [20.652,43.669] |  | Thyme | 26.356 | [21.056,31.656] |
|  |  |  |  | Butter | 36.168 | [23.225,49.111] |  | Butter | 21.123 | [16.875,25.370] |
|  |  |  |  | Ginger | 29.327 | [18.832,39.821] |  | Ginger | 23.259 | [18.582,27.936] |
|  |  |  |  | Butanol | 37.593 | [24.141,51.046] |  | Butanol | 23.345 | [18.651,28.040] |
|  |  |  | Adulthood (25-81) | | | | Scots |  |  |  |
|  |  |  |  | Banana | 28.288 | [22.507,34.068] |  | Banana | 27.808 | [22.612,33.005] |
|  |  |  |  | Peach | 25.028 | [19.914,30.143] |  | Peach | 28.558 | [23.221,33.895] |
|  |  |  |  | Cinnamon | 33.028 | [26.279,39.777] |  | Cinnamon | 31.722 | [25.794,37.650] |
|  |  |  |  | Jasmin | 22.613 | [17.992,27.234] |  | Jasmin | 26.856 | [21.838,31.875] |
|  |  |  |  | Strawberry | 28.909 | [23.002,34.817] |  | Strawberry | 33.335 | [27.105,39.564] |
|  |  |  |  | Fish | 29.197 | [23.231,35.163] |  | Fish | 25.185 | [20.479,29.892] |
|  |  |  |  | Garlic | 29.439 | [23.423,35.454] |  | Garlic | 26.826 | [21.813,31.84] |
|  |  |  |  | Thyme | 33.799 | [26.892,40.705] |  | Thyme | 29.276 | [23.805,34.747] |
|  |  |  |  | Butter | 33.078 | [26.319,39.838] |  | Butter | 27.726 | [22.545,32.908] |
|  |  |  |  | Ginger | 31.078 | [24.728,37.429] |  | Ginger | 26.146 | [21.260,31.032] |
|  |  |  |  | Butanol | 32.3 | [25.699,38.900] |  | Butanol | 31.771 | [25.834,37.708] |
|  |  |  | Pleasant vs unpleasant | | | |  |  |  |  |
|  |  |  |  | Pleasant | 27.001 | [24.318,29.684] |  |  |  |  |
|  |  |  |  | Unpleasant | 33.097 | [29.808,36.386] |  |  |  |  |

**Table S3**

Detailed results of the multilevel model with the following predictors included: Culture (with Dani as the reference group), age, type of odour (with Banana as the reference group) and their interaction, and odour familiarity.

| **Fixed effect** | **β** | **SE** | **95%CI** | **p** |
| --- | --- | --- | --- | --- |
| (Intercept) | 0.048 | 0.088 | [-0.125,0.221] | .587 |
| Age | 0.05 | 0.073 | [-0.093,0.193] | .491 |
| Culture [Hadza] | 0.641 | 0.119 | [ 0.408,0.873] | < .001*** |
| Culture [Pakistani] | 0.423 | 0.12 | [ 0.187,0.659] | < .001*** |
| Culture [Scots] | 0.165 | 0.117 | [-0.065,0.394] | .16 |
| Smell subscale factor [Peach] | 0.211 | 0.114 | [-0.013,0.434] | .065 |
| Smell subscale factor [Cinnamon] | 0.399 | 0.114 | [ 0.175,0.623] | < .001*** |
| Smell subscale factor [Jasmine] | 0.702 | 0.114 | [ 0.478,0.926] | < .001*** |
| Smell subscale factor [Strawberry] | 0.149 | 0.114 | [-0.075,0.373] | .193 |
| Smell subscale factor [Fish] | -0.981 | 0.114 | [-1.205, -0.757] | < .001*** |
| Smell subscale factor [Garlic] | -0.924 | 0.114 | [-1.148, -0.700] | < .001*** |
| Smell subscale factor [Thyme] | 0.141 | 0.114 | [-0.083,0.366] | .216 |
| Smell subscale factor [Butter] | -0.141 | 0.114 | [-0.365,0.083] | .217 |
| Smell subscale factor [Ginger] | -0.391 | 0.114 | [-0.615, -0.167] | < .001*** |
| Smell subscale factor [Butanol] | -0.672 | 0.115 | [-0.898, -0.446] | < .001*** |
| Smell value familiarity | 0.211 | 0.014 | [ 0.184,0.238] | < .001*** |
| Age × Culture [Hadza] | -0.232 | 0.114 | [-0.455, -0.010] | .041* |
| Age × Culture [Pakistani] | -0.135 | 0.121 | [-0.371,0.102] | .264 |
| Age × Culture [Scots] | -0.043 | 0.105 | [-0.248,0.162] | .68 |
| Age × Smell subscale factor [Peach] | 0.087 | 0.094 | [-0.098,0.272] | .358 |
| Age × Smell subscale factor [Cinnamon] | -0.052 | 0.094 | [-0.237,0.133] | .58 |
| Age × Smell subscale factor [Jasmine] | -0.048 | 0.094 | [-0.233,0.137] | .608 |
| Age × Smell subscale factor [Strawberry] | -0.053 | 0.094 | [-0.238,0.133] | .578 |
| Age × Smell subscale factor [Fish] | 0.053 | 0.094 | [-0.132,0.238] | .574 |
| Age × Smell subscale factor [Garlic] | -0.03 | 0.094 | [-0.215,0.155] | .754 |
| Age × Smell subscale factor [Thyme] | -0.1 | 0.094 | [-0.285,0.085] | .29 |
| Age × Smell subscale factor [Butter] | 0.052 | 0.094 | [-0.133,0.237] | .58 |
| Age × Smell subscale factor [Ginger] | -0.152 | 0.094 | [-0.337,0.033] | .107 |
| Age × Smell subscale factor [Butanol] | -0.147 | 0.094 | [-0.332,0.038] | .12 |
| Culture [Hadza] × Smell subscale factor [Peach] | -0.193 | 0.154 | [-0.494,0.109] | .21 |
| Culture [Pakistani] × Smell subscale factor [Peach] | -0.234 | 0.156 | [-0.539,0.072] | .133 |
| Culture [Scots] × Smell subscale factor [Peach] | -0.072 | 0.152 | [-0.370,0.225] | .634 |
| Culture [Hadza] × Smell subscale factor [Cinnamon] | -0.679 | 0.154 | [-0.980, -0.378] | < .001*** |
| Culture [Pakistani] × Smell subscale factor [Cinnamon] | -0.976 | 0.156 | [-1.282, -0.671] | < .001*** |
| Culture [Scots] × Smell subscale factor [Cinnamon] | -0.826 | 0.152 | [-1.125, -0.528] | < .001*** |
| Culture [Hadza] × Smell subscale factor [Jasmine] | -0.729 | 0.154 | [-1.030, -0.428] | < .001*** |
| Culture [Pakistani] × Smell subscale factor [Jasmine] | -0.532 | 0.156 | [-0.837, -0.226] | < .001*** |
| Culture [Scots] × Smell subscale factor [Jasmine] | -0.715 | 0.152 | [-1.013, -0.416] | < .001*** |
| Culture [Hadza] × Smell subscale factor [Strawberry] | -0.12 | 0.154 | [-0.421,0.181] | .434 |
| Culture [Pakistani] × Smell subscale factor [Strawberry] | -0.187 | 0.156 | [-0.493,0.118] | .229 |
| Culture [Scots] × Smell subscale factor [Strawberry] | 0.019 | 0.152 | [-0.278,0.317] | .899 |
| Culture [Hadza] × Smell subscale factor [Fish] | 0.064 | 0.154 | [-0.237,0.365] | .678 |
| Culture [Pakistani] × Smell subscale factor [Fish] | -0.481 | 0.156 | [-0.786, -0.175] | .002** |
| Culture [Scots] × Smell subscale factor [Fish] | -0.287 | 0.152 | [-0.585,0.010] | .059 |
| Culture [Hadza] × Smell subscale factor [Garlic] | 0.28 | 0.154 | [-0.022,0.581] | .069 |
| Culture [Pakistani] × Smell subscale factor [Garlic] | 0.004 | 0.156 | [-0.301,0.310] | .978 |
| Culture [Scots] × Smell subscale factor [Garlic] | 0.16 | 0.152 | [-0.137,0.457] | .291 |
| Culture [Hadza] × Smell subscale factor [Thyme] | -0.447 | 0.154 | [-0.748, -0.146] | .004** |
| Culture [Pakistani] × Smell subscale factor [Thyme] | -0.814 | 0.156 | [-1.120, -0.509] | < .001*** |
| Culture [Scots] × Smell subscale factor [Thyme] | -0.704 | 0.152 | [-1.001, -0.407] | < .001*** |
| Culture [Hadza] × Smell subscale factor [Butter] | -0.412 | 0.154 | [-0.713, -0.111] | .007** |
| Culture [Pakistani] × Smell subscale factor [Butter] | -1.065 | 0.156 | [-1.372, -0.759] | < .001*** |
| Culture [Scots] × Smell subscale factor [Butter] | -0.712 | 0.152 | [-1.010, -0.414] | < .001*** |
| Culture [Hadza] × Smell subscale factor [Ginger] | 0.309 | 0.154 | [ 0.007,0.610] | .045* |
| Culture [Pakistani] × Smell subscale factor [Ginger] | -0.468 | 0.156 | [-0.774, -0.162] | .003** |
| Culture [Scots] × Smell subscale factor [Ginger] | -0.467 | 0.152 | [-0.764, -0.169] | .002** |
| Culture [Hadza] × Smell subscale factor [Butanol] | 0.352 | 0.154 | [ 0.050,0.654] | .022* |
| Culture [Pakistani] × Smell subscale factor [Butanol] | 0.108 | 0.156 | [-0.197,0.413] | .488 |
| Culture [Scots] × Smell subscale factor [Butanol] | 0.272 | 0.152 | [-0.027,0.570] | .074 |
| (Age × Culture [Hadza]) × Smell subscale factor [Peach] | -0.05 | 0.147 | [-0.338,0.239] | .736 |
| (Age × Culture [Pakistani]) × Smell subscale factor [Peach] | -0.07 | 0.156 | [-0.377,0.236] | .652 |
| (Age × Culture [Scots]) × Smell subscale factor [Peach] | -0.023 | 0.136 | [-0.289,0.243] | .863 |
| (Age × Culture [Hadza]) × Smell subscale factor [Cinnamon] | 0.172 | 0.147 | [-0.116,0.461] | .242 |
| (Age × Culture [Pakistani]) × Smell subscale factor [Cinnamon] | 0.266 | 0.156 | [-0.041,0.572] | .09 |
| (Age × Culture [Scots]) × Smell subscale factor [Cinnamon] | -0.023 | 0.137 | [-0.291,0.245] | .866 |
| (Age × Culture [Hadza]) × Smell subscale factor [Jasmine] | 0.355 | 0.147 | [ 0.066,0.643] | .016* |
| (Age × Culture [Pakistani]) × Smell subscale factor [Jasmine] | -0.025 | 0.156 | [-0.331,0.282] | .875 |
| (Age × Culture [Scots]) × Smell subscale factor [Jasmine] | 0.044 | 0.136 | [-0.222,0.310] | .745 |
| (Age × Culture [Hadza]) × Smell subscale factor [Strawberry] | 0.112 | 0.147 | [-0.177,0.400] | .449 |
| (Age × Culture [Pakistani]) × Smell subscale factor [Strawberry] | 0.048 | 0.156 | [-0.259,0.354] | .761 |
| (Age × Culture [Scots]) × Smell subscale factor [Strawberry] | 0.104 | 0.136 | [-0.162,0.371] | .442 |
| (Age × Culture [Hadza]) × Smell subscale factor [Fish] | 0.122 | 0.147 | [-0.167,0.410] | .409 |
| (Age × Culture [Pakistani]) × Smell subscale factor [Fish] | -0.217 | 0.156 | [-0.523,0.089] | .165 |
| (Age × Culture [Scots]) × Smell subscale factor [Fish] | -0.11 | 0.136 | [-0.376,0.156] | .417 |
| (Age × Culture [Hadza]) × Smell subscale factor [Garlic] | 0.064 | 0.148 | [-0.226,0.354] | .665 |
| (Age × Culture [Pakistani]) × Smell subscale factor [Garlic] | 0.129 | 0.156 | [-0.178,0.435] | .411 |
| (Age × Culture [Scots]) × Smell subscale factor [Garlic] | 0.109 | 0.136 | [-0.157,0.375] | .421 |
| (Age × Culture [Hadza]) × Smell subscale factor [Thyme] | 0.129 | 0.147 | [-0.160,0.417] | .382 |
| (Age × Culture [Pakistani]) × Smell subscale factor [Thyme] | 0.151 | 0.156 | [-0.156,0.457] | .335 |
| (Age × Culture [Scots]) × Smell subscale factor [Thyme] | 0.121 | 0.136 | [-0.145,0.387] | .373 |
| (Age × Culture [Hadza]) × Smell subscale factor [Butter] | 0.037 | 0.147 | [-0.252,0.325] | .802 |
| (Age × Culture [Pakistani]) × Smell subscale factor [Butter] | 0.028 | 0.156 | [-0.278,0.335] | .856 |
| (Age × Culture [Scots]) × Smell subscale factor [Butter] | 0.037 | 0.136 | [-0.229,0.302] | .788 |
| (Age × Culture [Hadza]) × Smell subscale factor [Ginger] | 0.423 | 0.147 | [ 0.134,0.711] | .004** |
| (Age × Culture [Pakistani]) × Smell subscale factor [Ginger] | 0.234 | 0.156 | [-0.072,0.540] | .134 |
| (Age × Culture [Scots]) × Smell subscale factor [Ginger] | 0.187 | 0.136 | [-0.079,0.453] | .168 |
| (Age × Culture [Hadza]) × Smell subscale factor [Butanol] | 0.397 | 0.147 | [ 0.108,0.685] | .007** |
| (Age × Culture [Pakistani]) × Smell subscale factor [Butanol] | 0.009 | 0.156 | [-0.297,0.315] | .954 |
| (Age × Culture [Scots]) × Smell subscale factor [Butanol] | 0.027 | 0.136 | [-0.240,0.295] | .842 |

*Note*. * *p* < .05, ** *p* < .01, *** *p* < .001.

**Table S4**

Detailed results of the multilevel model without the interaction between Culture (with Dani as the reference group), Age, and type of odour (with Banana as the reference group), and odour familiarity.

| **Fixed effect** | **F** | **p** | **β** | **SE** | **95%CI** | **p** |
| --- | --- | --- | --- | --- | --- | --- |
| Intercept |  |  | 0.27 | 0.056 | [ 0.160,0.380] | < .001*** |
| Age | 0.885 | 0.347 | -0.019 | 0.02 | [-0.058,0.020] | 0.347 |
| Culture | 46.406 | < .001*** |  |  |  |  |
| Culture [Pakistani] |  |  | 0.464 | 0.058 | [ 0.351,0.577] | < .001*** |
| Culture [Dani] |  |  | 0.003 | 0.058 | [-0.111,0.117] | 0.959 |
| Culture [Scots] |  |  | -0.15 | 0.057 | [-0.261, -0.039] | 0.008 |
| Type of odour | 3880.3 | < .001*** |  |  |  |  |
| Type of odour [Peach] |  |  | 0.094 | 0.054 | [-0.012,0.200] | 0.083 |
| Type of odour [Cinnamon] |  |  | -0.232 | 0.054 | [-0.339, -0.126] | < .001*** |
| Type of odour [Jasmin] |  |  | 0.2 | 0.054 | [ 0.093,0.306] | < .001*** |
| Type of odour [Strawberry] |  |  | 0.079 | 0.054 | [-0.028,0.185] | 0.147 |
| Type of odour [Fish] |  |  | -1.135 | 0.054 | [-1.241, -1.028] | < .001*** |
| Type of odour [Garlic] |  |  | -0.796 | 0.055 | [-0.903, -0.689] | < .001*** |
| Type of odour [Thyme] |  |  | -0.36 | 0.055 | [-0.467, -0.253] | < .001*** |
| Type of odour [Butter] |  |  | -0.687 | 0.055 | [-0.794, -0.579] | < .001*** |
| Type of odour [Ginger] |  |  | -0.542 | 0.055 | [-0.650, -0.435] | < .001*** |
| Type of odour [Butanol] |  |  | -0.442 | 0.055 | [-0.551, -0.334] | < .001*** |
| Smell value familiarity | 319.0629 | < .001*** | 0.246 | 0.014 | [ 0.219,0.273] | < .001*** |
| **Random effects** | **Variance** | **SD** |  |  |  |  |
| Participant | 142.7 | 11.95 |  |  |  |  |
| Residual | 855.6 | 29.25 |  |  |  |  |
| **Model’s fit** |  |  |  |  |  |  |
| AIC | 41325.2 |  |  |  |  |  |
| BIC | 41439.6 |  |  |  |  |  |
| df | 4245 |  |  |  |  |  |
| ICC | 0.14 |  |  |  |  |  |
| Pseudo *r^2^* (fixed) | 0.34 |  |  |  |  |  |
| Pseudo *r^2^* (total) | 0.43 |  |  |  |  |  |

*Note*. * *p* < .05, ** *p* < .01, *** *p* < .001.

**Table S5**

Detailed results of the post-hoc analyses with Tukey correction.

| **Culture** | **Estimate** | **SE** | **p** |
| --- | --- | --- | --- |
| Dani - Hadza | -19.345 | 2.29 | <.001*** |
| Dani - Pakistani | -0.017 | 2.29 | 1 |
| Dani - Scots | 5.381 | 2.23 | 0.0763 |
| Hadza - Pakistani | 19.328 | 2.21 | <.001*** |
| Hadza - Scots | 24.726 | 2.14 | <.001*** |
| Pakistani - Scots | 5.398 | 2.14 | 0.0574 |
| **Type of odour** | **Estimate** | **SE** | **p** |
| Banana - Peach | -3.66 | 2.11 | .817 |
| Banana - Cinnamon | 9.02 | 2.12 | <.001*** |
| Banana - Jasmin | -7.76 | 2.11 | .011* |
| Banana - Strawberry | -3.06 | 2.11 | .935 |
| Banana - Fish | 44.1 | 2.11 | <.001*** |
| Banana - Garlic | 30.93 | 2.12 | <.001*** |
| Banana - Thyme | 13.99 | 2.12 | <.001*** |
| Banana - Butter | 26.67 | 2.14 | <.001*** |
| Banana - Ginger | 21.07 | 2.13 | <.001*** |
| Banana - Butanol | 17.18 | 2.15 | <.001*** |
| Peach - Cinnamon | 12.68 | 2.11 | <.001*** |
| Peach - Jasmin | -4.1 | 2.11 | .685 |
| Peach - Strawberry | 0.6 | 2.11 | 1 |
| Peach - Fish | 47.76 | 2.1 | <.001*** |
| Peach - Garlic | 34.59 | 2.11 | <.001*** |
| Peach - Thyme | 17.65 | 2.11 | <.001*** |
| Peach - Butter | 30.33 | 2.12 | <.001*** |
| Peach - Ginger | 24.73 | 2.11 | <.001*** |
| Peach - Butanol | 20.84 | 2.12 | <.001*** |
| Cinnamon - Jasmin | -16.79 | 2.11 | <.001*** |
| Cinnamon - Strawberry | -12.08 | 2.11 | <.001*** |
| Cinnamon - Fish | 35.08 | 2.1 | <.001*** |
| Cinnamon - Garlic | 21.91 | 2.1 | <.001*** |
| Cinnamon - Thyme | 4.96 | 2.1 | .393 |
| Cinnamon - Butter | 17.65 | 2.11 | <.001*** |
| Cinnamon - Ginger | 12.05 | 2.11 | <.001*** |
| Cinnamon - Butanol | 8.16 | 2.11 | .006** |
| Jasmine - Strawberry | 4.7 | 2.11 | .482 |
| Jasmine - Fish | 51.86 | 2.11 | <.001*** |
| Jasmine - Garlic | 38.7 | 2.11 | <.001*** |
| Jasmine - Thyme | 21.75 | 2.11 | <.001*** |
| Jasmine - Butter | 34.43 | 2.12 | <.001*** |
| Jasmine - Ginger | 28.83 | 2.12 | <.001*** |
| Jasmine - Butanol | 24.94 | 2.13 | <.001*** |
| Strawberry - Fish | 47.16 | 2.11 | <.001*** |
| Strawberry - Garlic | 33.99 | 2.11 | <.001*** |
| Strawberry - Thyme | 17.05 | 2.11 | <.001*** |
| Strawberry - Butter | 29.73 | 2.12 | <.001*** |
| Strawberry - Ginger | 24.13 | 2.12 | <.001*** |
| Strawberry - Butanol | 20.24 | 2.13 | <.001*** |
| Fish - Garlic | -13.16 | 2.1 | <.001*** |
| Fish - Thyme | -30.11 | 2.1 | <.001*** |
| Fish - Butter | -17.43 | 2.11 | <.001*** |
| Fish - Ginger | -23.03 | 2.11 | <.001*** |
| Fish - Butanol | -26.92 | 2.12 | <.001*** |
| Garlic - Thyme | -16.95 | 2.1 | <.001*** |
| Garlic - Butter | -4.26 | 2.11 | .631 |
| Garlic - Ginger | -9.86 | 2.1 | <.001*** |
| Garlic - Butanol | -13.76 | 2.11 | <.001*** |
| Thyme - Butter | 12.68 | 2.1 | <.001*** |
| Thyme - Ginger | 7.08 | 2.1 | .031* |
| Thyme - Butanol | 3.19 | 2.11 | .915 |
| Butter - Ginger | -5.6 | 2.1 | .218 |
| Butter - Butanol | -9.49 | 2.11 | <.001*** |
| Ginger - Butanol | -3.89 | 2.11 | .752 |

**Table S6**

Spearman’s rank correlation coefficients for the mean pleasantness ratings of the 11 odours for each pair of cultures.

| **Culture** | **Hadza** | **Pakistani** | **Dani** | **Scots** |
| --- | --- | --- | --- | --- |
| Hadza | 1 |  |  |  |
| Pakistani | 0.861*** | 1 |  |  |
| Dani | 0.697* | 0.772** | 1 |  |
| Scots | 0.842** | 0.882*** | 0.655* | 1 |

*Note*. * *p* < .05, ** *p* < .01, *** *p* < .001.

**Table S7**

Residual variability across the six age groups (the lower the score, the higher the similarity in odour pleasantness ratings; conversely, the higher the score, the higher the variability in odour pleasantness ratings).

| **Age group** | **Variability** | **95%CI** |
| --- | --- | --- |
| 3-4 | 43.279 | [38.219,48.304] |
| 5-6 | 35.508 | [31.356,39.660] |
| 7-8 | 35.558 | [31.400,39.716] |
| 9-10 | 34.121 | [30.132,38.111] |
| 11-12 | 34.236 | [30.233,38.240] |
| 13-15 | 29.529 | [26.076,32.981] |

**Table S8**

Average odour pleasantness ratings (POMP scores) in the full sample, four age subgroups, and four cultures (Dani, Hadza, Pakistani, Scots) with excluded data from children younger than 5 years (*n* = 10) and adults older than 60 years (*n* = 3).

|  | **Full sample** | **Early childhood (3-7)** | **Middle childhood (8-12)** | **Adolescence/emerging adulthood (13-24)** | **Adulthood (25-81)** | **Hadza** | **Pakistani** | **Dani** | **Scots** |
| --- | --- | --- | --- | --- | --- | --- | --- | --- | --- |
| **Odour** | **Mean (SD)** | **Mean (SD)** | **Mean (SD)** | **Mean (SD)** | **Mean (SD)** | **Mean (SD)** | **Mean (SD)** | **Mean (SD)** | **Mean (SD)** |
| Banana | 71.63 (29.98) | 66.38 (31.86) | 75.29 (26.98) | 79.17 (28.68) | 67.22 (32.81) | 85 (32.95) | 76.58 (19.23) | 58.23 (32.64) | 64.6 (26.52) |
| Peach | 72.49 (30.65) | 63.07 (35.15) | 75.58 (29.2) | 79.17 (25.5) | 73.61 (28.63) | 86.5 (32.45) | 72.63 (20.33) | 66.46 (33.61) | 63.48 (29.78) |
| Cinnamon | 58.22 (37.17) | 54.02 (35.12) | 57.6 (38.36) | 60 (39.72) | 62.92 (35.98) | 72.5 (42.27) | 47.11 (32.59) | 71.65 (27.15) | 43.5 (34.01) |
| Jasmine | 77.59 (29.14) | 77.56 (27.86) | 76.92 (29.88) | 70.83 (37.76) | 81.11 (25.5) | 85.5 (32.79) | 81.84 (21.09) | 85.67 (21.17) | 59 (29.63) |
| Strawberry | 73.28 (30.58) | 69.32 (32.1) | 78.38 (28.12) | 66.67 (34.32) | 69.72 (31.24) | 87 (31.48) | 73.68 (21.38) | 61.59 (27.55) | 68.81 (34.35) |
| Fish | 24.01 (34.70) | 21.02 (29.09) | 26.02 (36.72) | 20.83 (34.79) | 24.17 (36.03) | 48.5 (47.91) | 13.95 (22.1) | 17.38 (22.46) | 14.71 (24.21) |
| Garlic | 35.74 (36.29) | 28.74 (30.86) | 40.5 (38.55) | 31.67 (34.7) | 34.83 (36.47) | 60.1 (46.28) | 32.37 (27.01) | 18.9 (25.63) | 28.71 (27.47) |
| Thyme | 52.57 (38.12) | 45.45 (36.66) | 55.41 (38.97) | 51.67 (39.9) | 54.44 (37.02) | 71.5 (42.79) | 43.16 (28.35) | 59.45 (38.62) | 37.25 (31.42) |
| Butter | 38.49 (39.64) | 34.66 (36.44) | 38.53 (41.52) | 26.67 (38.24) | 46.11 (38.57) | 62.5 (46.26) | 18.68 (23.62) | 50 (37.06) | 24.01 (29.98) |
| Ginger | 45.50 (37.62) | 30.46 (31.99) | 48.25 (39.54) | 62.5 (32) | 49.17 (36.61) | 84.5 (33.1) | 33.16 (24.85) | 39.02 (28.35) | 23.76 (29.02) |
| Butanol | 47.81 (37.46) | 45.4 (35.87) | 51.32 (37.5) | 44.17 (38.66) | 44.66 (38.52) | 72.5 (42.86) | 45.53 (25.78) | 22.56 (26.53) | 46 (33.66) |

**Table S9**

Residual variability across the smells, cultures, and age group (the lower the score, the higher the similarity in odour pleasantness ratings; conversely, the higher the score, the higher the variability in odour pleasantness ratings) with excluded data from children younger than 5 years (*n* = 10) and adults older than 60 years (*n* = 3).

| **Odour** | **Variability** | **95%CI** | **Group** | **Odour** | **Variability** | **95%CI** | **Culture** | **Odour** | **Variability** | **95%CI** |
| --- | --- | --- | --- | --- | --- | --- | --- | --- | --- | --- |
| Banana | 2.659 | [21.381,31.805] | Early childhood (3-7) | | | | Hadza |  |  |  |
| Peach | 2.726 | [21.92,32.608] |  | Banana | 29.314 | [23.154,35.474] |  | Banana | 26.645 | [21.422,31.867] |
| Cinnamon | 3.282 | [26.388,39.254] |  | Peach | 32.904 | [25.990,39.819] |  | Peach | 28.552 | [22.956,34.148] |
| Jasmin | 2.61 | [20.987,31.219] |  | Cinnamon | 30.996 | [24.483,37.509] |  | Cinnamon | 37.128 | [29.851,44.405] |
| Strawberry | 2.792 | [22.447,33.391] |  | Jasmin | 23.308 | [18.410,28.205] |  | Jasmin | 29.028 | [23.339,34.718] |
| Fish | 2.856 | [22.962,34.158] |  | Strawberry | 30.2 | [23.854,36.546] |  | Strawberry | 27.889 | [22.423,33.356] |
| Garlic | 2.992 | [24.053,35.78] |  | Fish | 25.893 | [20.452,31.334] |  | Fish | 40.868 | [32.858,48.878] |
| Thyme | 3.308 | [26.598,39.566] |  | Garlic | 28.128 | [22.217,34.038] |  | Garlic | 38.761 | [31.164,46.359] |
| Butter | 3.442 | [27.676,41.169] |  | Thyme | 32.893 | [25.981,39.805] |  | Thyme | 37.128 | [29.851,44.405] |
| Ginger | 3.11 | [25.004,37.194] |  | Butter | 33.069 | [26.120,40.017] |  | Butter | 40.826 | [32.824,48.827] |
| Butanol | 3.252 | [26.144,38.89] |  | Ginger | 28.346 | [22.390,34.303] |  | Ginger | 30.634 | [24.63,36.639] |
|  |  |  |  | Butanol | 33.967 | [26.829,41.105] |  | Butanol | 37.723 | [30.329,45.117] |
|  |  |  | Middle childhood (8-12) | | | | Dani |  |  |  |
|  |  |  |  | Banana | 23.84 | [20.267,27.414] |  | Banana | 31.873 | [25.057,38.69] |
|  |  |  |  | Peach | 25.535 | [21.707,29.362] |  | Peach | 32.582 | [25.615,39.55] |
|  |  |  |  | Cinnamon | 32.527 | [27.651,37.402] |  | Cinnamon | 26.705 | [20.994,32.416] |
|  |  |  |  | Jasmin | 27.853 | [23.678,32.028] |  | Jasmin | 20.284 | [15.946,24.622] |
|  |  |  |  | Strawberry | 25.128 | [21.362,28.894] |  | Strawberry | 26.7 | [20.99,32.41] |
|  |  |  |  | Fish | 30.203 | [25.676,34.730] |  | Fish | 21.599 | [16.98,26.218] |
|  |  |  |  | Garlic | 30.538 | [25.961,35.115] |  | Garlic | 24.92 | [19.591,30.25] |
|  |  |  |  | Thyme | 33.055 | [28.101,38.010] |  | Thyme | 38.115 | [29.964,46.266] |
|  |  |  |  | Butter | 34.7 | [29.499,39.901] |  | Butter | 36.66 | [28.82,44.5] |
|  |  |  |  | Ginger | 30.757 | [26.147,35.367] |  | Ginger | 27.779 | [21.839,33.72] |
|  |  |  |  | Butanol | 30.804 | [26.187,35.421] |  | Butanol | 25.892 | [20.355,31.429] |
|  |  |  | Adolescence/emerging adulthood (13-24) | | | | Pakistani |  |  |  |
|  |  |  |  | Banana | 23.086 | [14.825,31.347] |  | Banana | 18.046 | [25.057,38.69] |
|  |  |  |  | Peach | 20.948 | [13.452,28.445] |  | Peach | 19.144 | [25.615,39.55] |
|  |  |  |  | Cinnamon | 38.832 | [24.936,52.728] |  | Cinnamon | 30.309 | [20.994,32.416] |
|  |  |  |  | Jasmin | 30.413 | [19.530,41.296] |  | Jasmin | 19.834 | [15.946,24.622] |
|  |  |  |  | Strawberry | 29.523 | [18.959,40.088] |  | Strawberry | 20.973 | [20.99,32.41] |
|  |  |  |  | Fish | 26.367 | [16.932,35.802] |  | Fish | 19.891 | [16.98,26.218] |
|  |  |  |  | Garlic | 31.309 | [20.105,42.513] |  | Garlic | 24.816 | [19.591,30.25] |
|  |  |  |  | Thyme | 32.16 | [20.652,43.669] |  | Thyme | 26.356 | [29.964,46.266] |
|  |  |  |  | Butter | 36.168 | [23.225,49.111] |  | Butter | 21.123 | [28.82,44.5] |
|  |  |  |  | Ginger | 29.327 | [18.832,39.821] |  | Ginger | 23.259 | [21.839,33.72] |
|  |  |  |  | Butanol | 37.593 | [24.141,51.046] |  | Butanol | 23.345 | [20.355,31.429] |
|  |  |  | Adulthood (25-81) | | | | Scots |  |  |  |
|  |  |  |  | Banana | 28.584 | [22.646,34.523] |  | Banana | 25.327 | [20.594,30.06] |
|  |  |  |  | Peach | 25.189 | [19.955,30.422] |  | Peach | 27.893 | [22.68,33.105] |
|  |  |  |  | Cinnamon | 33.084 | [26.211,39.958] |  | Cinnamon | 30.255 | [24.601,35.909] |
|  |  |  |  | Jasmin | 22.717 | [17.997,27.437] |  | Jasmin | 26.07 | [21.198,30.942] |
|  |  |  |  | Strawberry | 29.148 | [23.092,35.204] |  | Strawberry | 32.74 | [26.621,38.858] |
|  |  |  |  | Fish | 28.721 | [22.754,34.689] |  | Fish | 23.116 | [18.796,27.436] |
|  |  |  |  | Garlic | 29.218 | [23.148,35.289] |  | Garlic | 24.883 | [20.233,29.533] |
|  |  |  |  | Thyme | 33.743 | [26.732,40.753] |  | Thyme | 28.103 | [22.851,33.355] |
|  |  |  |  | Butter | 32.9 | [26.065,39.736] |  | Butter | 27.075 | [22.015,32.134] |
|  |  |  |  | Ginger | 30.78 | [24.385,37.175] |  | Ginger | 25.675 | [20.877,30.473] |
|  |  |  |  | Butanol | 31.927 | [25.294,38.561] |  | Butanol | 31.411 | [25.541,37.281] |
|  |  |  | Pleasant vs unpleasant | | | |  |  |  |  |
|  |  |  |  | Pleasant | 27.001 | [24.318,29.684] |  |  |  |  |
|  |  |  |  | Unpleasant | 33.097 | [29.808,36.386] |  |  |  |  |

**Table S10**

Detailed results of the multilevel model with the interaction between Culture (with Dani as the reference group) and the most vs least pleasant odours, Age, and odour familiarity.

| **Fixed effect** | **F** | ***p*** | **β** | **SE** | **95%CI** | ***p*** |
| --- | --- | --- | --- | --- | --- | --- |
| Intercept |  |  | -0.584 | 0.056 | [-0.695, -0.474] | < .001*** |
| Age | 0.034 | .854 | 0.004 | 0.021 | [-0.038,0.046] | .854 |
| Culture | 35.263 | <.001*** |  |  |  |  |
| Society [Hadza] | |  | 0.603 | 0.077 | [ 0.452,0.754] | < .001 |
| Society [Pakistani] | |  | -0.115 | 0.077 | [-0.267,0.036] | .136 |
| Society [Scots] | |  | -0.136 | 0.075 | [-0.283,0.010] | .069 |
| Most pleasant vs unpleasant | 1032.849 | <.001*** | 1.017 | 0.066 | [ 0.888,1.145] | < .001*** |
| Familiarity | 79.012 | <.001*** | 0.159 | 0.018 | [ 0.124,0.194] | < .001 |
| Culture x Most vs least pleasant | 13.097 | <.001*** |  |  |  |  |
| Culture [Hadza] × Most vs least pleasant [Pleasant] | | | -0.313 | 0.088 | [-0.487, -0.140] | < .001*** |
| Culture [Pakistani] × Most vs least pleasant [Pleasant] | | | 0.22 | 0.090 | [ 0.044,0.396] | .014* |
| Culture [Scots] × Most vs least pleasant [Pleasant] | | | 0.011 | 0.087 | [-0.160,0.182] | .903 |
| **Random effects** | **Variance** | **SD** |  |  |  |  |
| Participant | 133 | 11.53 |  |  |  |  |
| Residual | 853.6 | 29.22 |  |  |  |  |
| **Model’s fit** |  |  |  |  |  |  |
| AIC | 22580.24 |  |  |  |  |  |
| BIC | 22649.26 |  |  |  |  |  |
| df | 2314 |  |  |  |  |  |
| ICC | 0.13 |  |  |  |  |  |
| Pseudo *r^2^* (fixed) | 0.38 |  |  |  |  |  |
| Pseudo *r^2^* (total) | 0.46 |  |  |  |  |  |

*Note*. * *p* < .05, ** *p* < .01, *** *p* < .001.

**Table S11**

Detailed results of the multilevel model with the interaction between Culture (with Dani as the reference group) and type of odours, and odour familiarity, and age.

| **Fixed effect** | **F** | **p** | **β** | **SE** | **95%CI** | **p** |
| --- | --- | --- | --- | --- | --- | --- |
| Intercept |  |  | 0.23 | 0.058 | [0.116,0.344] | < .001*** |
| Age | 0.598 | .439 | -0.015 | 0.02 | [-0.054,0.024] | .440 |
| Culture | 1.175 | .318 |  |  |  |  |
| Culture [Hadza] | | | 0.402 | 0.059 | [ 0.286, 0.518] | < .001*** |
| Culture [Pakistani] | | | 0.006 | 0.058 | [-0.108, 0.121] | .913 |
| Culture [Scots] | | | -0.143 | 0.057 | [-0.254, -0.032] | .012* |
| Type of odour | 15.678 | <.001*** |  |  |  |  |
| Type of odour [Peach] | | | 0.134 | 0.057 | [ 0.022, 0.246] | .019* |
| Type of odour [Cinnamon] | | | -0.191 | 0.057 | [-0.303, -0.080] | < .001*** |
| Type of odour [Jasmin] | | | 0.253 | 0.057 | [ 0.140, 0.365] | < .001*** |
| Type of odour [Strawberry] | | | 0.109 | 0.058 | [-0.004, 0.222] | .058 |
| Type of odour [Fish] | | | -1.084 | 0.057 | [-1.195, -0.973] | < .001*** |
| Type of odour [Garlic] | | | -0.771 | 0.057 | [-0.882, -0.659] | < .001*** |
| Type of odour [Thyme] | | | -0.319 | 0.057 | [-0.430, -0.207] | < .001*** |
| Type of odour [Butter] | | | -0.642 | 0.058 | [-0.756, -0.529] | < .001*** |
| Type of odour [Ginger] | | | -0.502 | 0.057 | [-0.615, -0.389] | < .001*** |
| Type of odour [Butanol] | | | -0.411 | 0.059 | [-0.526, -0.295] | < .001*** |
| Familiarity | 144.366 | <.001*** | 0.396 | 0.104 | [0.192,0.600] | < .001 |
| Familiarity x Culture | 6.193 | <.001*** |  |  |  |  |
| Familiarity × Culture [Hadza] |  |  | 0.152 | 0.041 | [0.072,0.233] | < .001*** |
| Familiarity × Culture [Pakistani] |  |  | 0.017 | 0.038 | [-0.057,0.091] | .651 |
| Familiarity × Culture [Scots] |  |  | 0.004 | 0.038 | [-0.070,0.079] | .91 |
| Familiarity x Type of odour | 4.786 | <.001*** |  |  |  |  |
| Familiarity × Type of odour [Peach] |  |  | -0.151 | 0.061 | [-0.272,-0.031] | .014* |
| Familiarity × Type of odour [Cinnamon] |  |  | -0.073 | 0.059 | [-0.189,0.043] | .215 |
| Familiarity × Type of odour [Jasmine] |  |  | -0.199 | 0.06 | [-0.317,-0.080] | .001** |
| Familiarity × Type of odour [Strawberry] |  |  | -0.089 | 0.062 | [-0.210,0.032] | .149 |
| Familiarity × Type of odour [Fish] |  |  | -0.282 | 0.059 | [-0.398,-0.166] | < .001*** |
| Familiarity × Type of odour [Garlic] |  |  | -0.146 | 0.06 | [-0.263,-0.029] | .014* |
| Familiarity × Type of odour [Thyme] |  |  | -0.06 | 0.06 | [-0.177,0.057] | .312 |
| Familiarity × Type of odour [Butter] |  |  | -0.054 | 0.059 | [-0.171,0.062] | .36 |
| Familiarity × Type of odour [Ginger] |  |  | 0.014 | 0.06 | [-0.103,0.132] | .812 |
| Familiarity × Type of odour [Butanol] |  |  | -0.087 | 0.059 | [-0.203,0.029] | .142 |
| Familiarity × Age | 0.209 | .647 | 0.006 | 0.014 | [-0.021,0.033] | .647 |
| **Random effects** | **Variance** | **SD** |  |  |  |  |
| Participant | 145.1 | 12.05 |  |  |  |  |
| Residual | 841.4 | 29.01 |  |  |  |  |
| **Model’s fit** |  |  |  |  |  |  |
| AIC | 41286.1 |  |  |  |  |  |
| BIC | 41489.6 |  |  |  |  |  |
| df | 4231 |  |  |  |  |  |
| ICC | 0.15 |  |  |  |  |  |
| Pseudo *r^2^* (fixed) | 0.35 |  |  |  |  |  |
| Pseudo *r^2^* (total) | 0.44 |  |  |  |  |  |

*Note*. * *p* < .05, ** *p* < .01, *** *p* < .001.

**Figure S1**

*Similarity in odour pleasantness ratings across the six age groups (the lower the score, the higher the similarity in odour pleasantness ratings; conversely, the higher the score, the higher the variability in odour pleasantness ratings).*

*Note*. Error bars represent 95% confidence intervals.

**Figure S2**

*Similarity in odour pleasantness ratings across top three most pleasant (Jasmine, Strawberry, Peach) and unpleasant (Fish, Garlic, Butter) odours (the lower the score, the higher the similarity in odour pleasantness ratings; conversely, the higher the score, the higher the variability in odour pleasantness ratings).*

*Note*. Error bars represent 95% confidence intervals.
